# Five Unprecedented Secondary Metabolites from the Spider Parasitic Fungus *Akanthomyces novoguineensis*

**DOI:** 10.3390/molecules22060991

**Published:** 2017-06-14

**Authors:** Soleiman E. Helaly, Wilawan Kuephadungphan, Souwalak Phongpaichit, Janet Jennifer Luangsa-ard, Vatcharin Rukachaisirikul, Marc Stadler

**Affiliations:** 1 Department of Microbial Drugs, Helmholtz Centre for Infection Research, 38124 Braunschweig, Germany; soleiman.helaly@aswu.edu.eg (S.E.H.); wilawan.kue@hotmail.co.th (W.K.); 2 Department of Chemistry, Faculty of Science, Aswan University, Aswan 81528, Egypt; 3 Department of Microbiology, Faculty of Science, Prince of Songkla University, Songkhla 90112, Thailand; souwalak.p@psu.ac.th; 4 Natural Products Research Center of Excellence and Department of Microbiology, Prince of Songkla University, Songkhla 90112, Thailand; 5 National Centre for Genetic Engineering and Biotechnology (BIOTEC), Pathumthani 12120, Thailand; jajen@biotec.or.th; 6 Department of Chemistry, Faculty of Science, Prince of Songkla University, Songkhla 90112, Thailand; vatcharin.r@psu.ac.th; 7 Center of Excellence for Innovation in Chemistry, Prince of Songkla University, Songkhla 90112, Thailand

**Keywords:** akanthol, akanthozine, *Akanthomyces novoguineensis*, hydroxamic acid, chemotaxonomy

## Abstract

Five new compounds including the glycosylated β-naphthol (**1**, akanthol), a glycosylated pyrazine (**2**, akanthozine), and three amide derivatives including a hydroxamic acid derivative (**3**–**5**) were isolated from the spider-associated fungus *Akanthomyces novoguineensis* (Cordycipitaceae, Ascomycota). Their structures were elucidated by using high resolution mass spectrometry (HRMS) and NMR spectroscopy. In this study, the antimicrobial, cytotoxic, anti-biofilm, and nematicidal activities of the new compounds were evaluated. The distribution pattern of secondary metabolites in the species was also revealed in which more isolates of *A. novoguineensis* were encountered and their secondary metabolite profiles were examined using analytical HPLC with diode array and mass spectrometric detection (HPLC-DAD/MS). Remarkably, all isolated compounds are specifically produced by *A. novoguineensis*.

## 1. Introduction

For over decades, fungi that are closely associated with invertebrates are accepted as a rich source for a wide range of bioactive secondary metabolites [1,2]. The largest numbers of invertebrate-pathogenic fungi belong to the order Hypocreales in which many species have been investigated for a long time and have proven to be effective biocontrol agents as well as prolific producers of biologically active secondary metabolites [2,3]. So far, certain ecological groups of hypocrealean fungi, and in particular the parasites of spiders have not yet received much attention, despite the fact that their close phylogenetic relatives have been proven to be an attractive source of natural products. In a concurrent study, four unprecedented glycosylated α-pyrone derivatives, akanthopyrones A–D, were obtained from a culture of *A. novoguineensis* [4]. These findings lead us to further investigate this species and its allies. As a part of our ongoing research on biologically active fungal metabolites [5], the constituents of several *A. novoguineensis* isolates were compared. Scale-up fermentation and subsequent extensive chromatography of the organic crude extracts led to the isolation of five new compounds. Herein, we report on the isolation, structure elucidation, biological activities of the new compounds (**1**–**5**), and the species-specific pattern of secondary metabolite production in *A. novoguineensis*.

## 2. Results and Discussion

**Figure 1:**
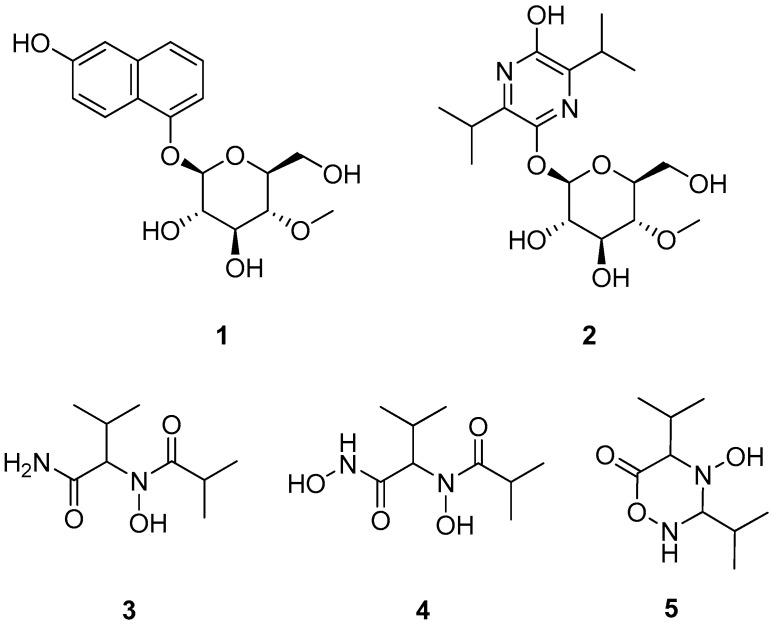
Structures of Compounds **1**–**5**.

Compounds **1**–**3** were isolated from the ethyl acetate extract of *A. novoguineensis* BCC47894 as described in the experimental section, while Compounds **4** and **5** were obtained from the ethyl acetate extract of *A. novoguineensis* BCC47881 (Figure 1).

**Figure 2:**
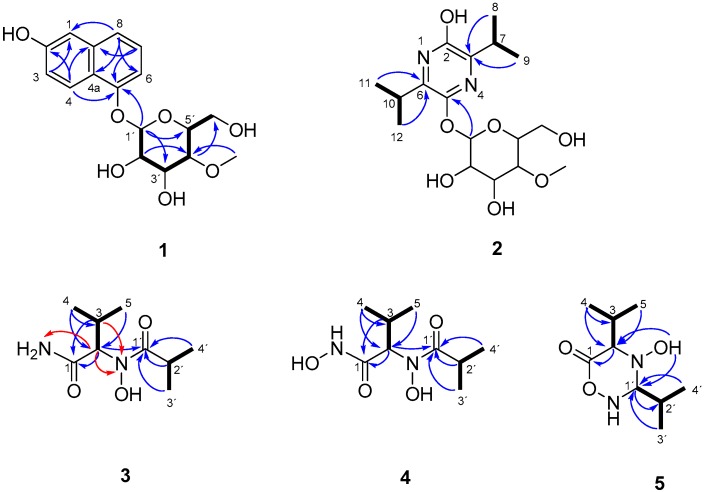
Key COSY (bold bonds), ^13^C-HMBC (blue arrows), and ^15^N-HMBC (red arrows) correlations for Compounds **1**–**5**.

Compound **1** was obtained as a pale brown gum. HRMS afforded the molecular ion cluster at *m*/*z* 359.1107 [M + Na]^+^, which furnished the molecular formula of C_17_H_28_O_7_ (calcd. for [M + Na]^+^ 359.1101). The ^1^H- and ^13^C-NMR data revealed the presence of six *sp*^2^ olefinic carbons, five *sp*^3^ methine carbons, one of which is anomeric carbon (δ_H_ 5.07, δ_C_ 102.5), hence suggesting the presence of sugar moiety in **1**, one methylene carbon, one methoxy carbon, and four *sp*^2^ quaternary carbons. The sugar moiety was assigned as 4-*O*-methyl-glucopyranose based on COSY and HMBC spectra. In addition to the sugar signals, only signals in the aromatic region were observed. Thus, the aglycone was determined by COSY correlation between 3-H/4-H and between 6-H/7-H/8-H together with a series of HMBC correlations from 8-H to C-1/C-4a/C-6, from 6-H to C-4/C-4a/C-8, from 7-H to C-5/C-8a, and from 4-H to C-2/C-8a/C-5 allowed the construction of a 2-naphthol substructure (Figure 2). Finally, the glycosidic linkage at C-5 was evident by HMBC correlation from the anomeric methine (H-1′) to C-5.

The relative configuration of the sugar moiety in **1** was determined by NOESY data and coupling constant analysis. Starting from a characteristic H-1′ signal of a β-glycoside at δ_H_ 5.07 with *J*_1′,2′_ = 8.2 Hz a chain of vicinal *trans* couplings of *J*_2′,3′_ = 8.6 Hz, *J*_3′,4′_ = 8.6 Hz, and *J*_4′,5′_ = 9.5 Hz supported by NOEs between H-1′ and H-3′/H-5′ determined the configuration of the sugar moiety as 4-*O*-methyl-β-glucopyranose. Its β-glycoside connection was evident by a ^1^*J*_C,H_ coupling of 162 Hz for the anomeric methine. The d-configuration of the sugar was established by comparing the specific rotation of the aqueous layer of its acid hydrolysate ${\left[\alpha \right]}_{D}^{25}$ +36, *c* 0.05, MeOH) with that of 4-*O*-methyl-d-glucopyranose ${\left[\alpha \right]}_{D}^{25}$ +80, *c* 1.3, MeOH) [6]. Consequently, Compound **1** was determined as the new natural product, β-naphthol bearing 4-*O*-methyl-β-d-glucopyranose, for which we have proposed the trivial name akanthol.

Akanthozine (**2**) possesses the molecular formula of C_17_H_28_N_2_O_7_ as determined from the molecular ion peak at *m*/*z* 373.1971 [M + H]^+^ (calcd. 373.1969). The sugar moiety signals were preserved in the NMR data of **2**. In addition, the remaining 10 carbon signals were determined as follow: four douplets assigned to two sets of germinal methyls, two methines, and four *sp*^2^ quaternary carbons. COSY spectra showed correlations between the germinal methyls H_3_-8/H_3_-9 and the methine H-7 and between the germinal methyls H_3_-11/H_3_-12 and the methine H-10. Furthermore, HMBC correlations from the germinal methyls H_3_-8/H_3_-9 to C-3, from H_3_-11/H-12 to C-6, and from H-7 to C-2, together with the two nitrogen atoms, implied by the molecular formula, determined the agylcone as 3,6-diisopropylpyrazine-2-ol (Figure 2). Finally, HMBC correlation from the anomeric proton H-1′ to C-5 established the glycosidic bond in **2**. Similarly to **1**, NOESY data and coupling constant values for **2** determined the sugar unit as 4-*O*-methyl-β-d-glucopyranose. The co-occurrence and the positive specific rotation value of **2** ${\left[\alpha \right]}_{D}^{25}$ +20°, *c* 0.17, MeOH) suggested that the sugar in **2** possesses the same absolute d-configuration as **1**. Consequently, Compound **2** was determined as a new natural product, 3,6-diisopropylpyrazine-2-ol bearing 4-*O*-methyl-β-d-glucopyranose and was named akanthozine.

**Figure 3:**
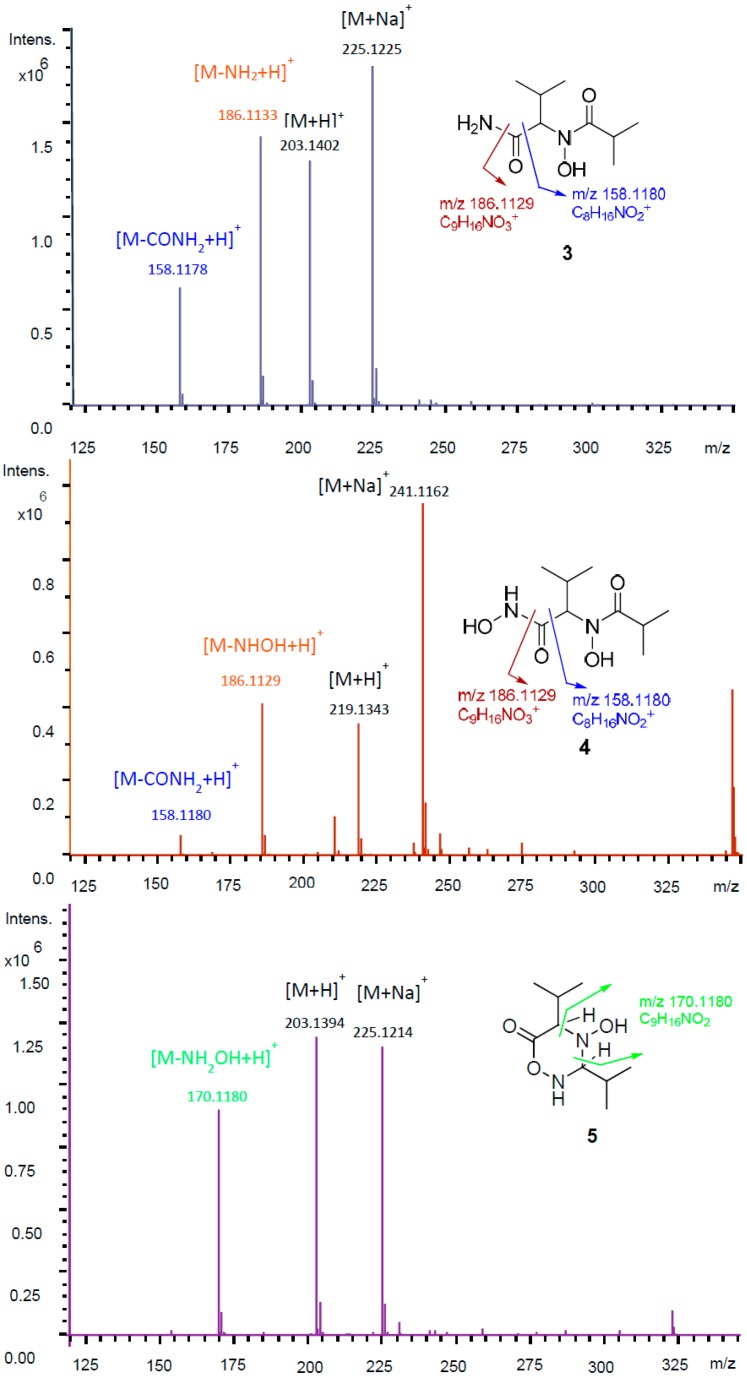
HRMS spectra for Compounds **3**–**5**.

Compound **3** has the molecular formula of C_9_H_18_N_2_O_3_ as determined from the molecular in peak at *m*/*z* 203.1389 [M + H]^+^ (calcd. 203.1390) with two unsaturation degrees. The relatively small molecular weight suggested the lack of the sugar moiety in **3**. ^13^C- and HSQC NMR data revealed the presence of four methyls, three methines, and two carbonyls, which is required for the unsaturation degrees, so Compound **3** must be acyclic. COSY and HMBC data established two substructures 3-methylbutanamide and *N*-hydroxyisobutyramide as shown in Figure 2. An HMBC correlation from the methine 2-H (δ_H_ 4.60, δ_C_ 65.8) to the carbonyl carbon 1′-C established the linkage of the *N*-hydroxyisobutyramide at C-2 of the 3-methylbutanamide by a C–N bond. Furthermore, ^1^H-^15^N HSQC and ^1^H-^15^N HMBC experiments were performed in order to confirm the structure of **3**, which showed correlations from 2-H/3-H to the tertiary amide nitrogen (δ_N_ 177.2) and from 2-H to the primary amide nitrogen (δ_N_ 106.4). Additionally, HR-ESI-MS spectra of **3** showed two characteristic fragments at *m*/*z* 186.1133 (C_9_H_16_NO_3_; assigned to the loss of NH_2_ group) and at *m*/*z* 158.1178 (C_8_H_16_NO_2_, attributed to the cleavage of amide group (CONH_2_). This further confirmed the structure of **3** (Figure 3). Consequently, Compound **3** was determined as new butanamide derivative, namely 2-(*N*-hydroxyisobutyramide)-3-methylbutanamide. To the best of our knowledge, this is the first report on this compound.

Compound **4** was obtained as a white amorphous powder; it showed the molecular formula of C_9_H_18_N_2_O_4_ calculated from the ion peak at *m*/*z* 219.1343 [M + H]^+^ (calcd. 219.1339). At first glance, Compound **4** differs from **3** by the presence of an additional oxygen atom. The ^1^H- and ^13^C-NMR data of **4** revealed a high similarity to those of **3**; nevertheless, slight shifts were observed. Particularly, in the ^13^C-NMR spectrum, the carbonyl carbon C-1 was shifted from δ_C_ 175.2 in **3** to δ_C_ 168.9 in **4** suggested that the additional oxygen could be attached to the amide group (C-1). The 2D NMR data for **4** showed no further remarkable differences to those of **3**. Nevertheless, the HRMS spectrum of **4** showed a different fragmentation pattern to that of **3**. A fraction ion peak at *m/z* 186.1129 (C_9_H_16_NO_3_) was assigned to the cleavage of hydroxyamide (NHOH) and a fraction peak at *m/z* 158.1180 (C_8_H_16_NO_2_) was assigned to the cleavage of hydroxamic group (CONHOH) (Figure 3). This was supported by the singlet band in the IR spectrum at 3466 cm^−1^ (secondary amine). Thus, the structure for **4** was determined as a new hydroxamic acid derivative, namely *N*-hydroxy-2-(*N*-hydroxyisobutyramido)-3-methylbutanamide. The newly released ACD labs’ Structure elucidator was used to confirm the suggested structure of **4**; the structure showed the lowest Δδ_C_ (δ_Experimental_ − δ_Calculated_) d_N_^13^C = 1.34 and thus was assigned as the best structure among the 54 generated structures by the software [7].

Compound **5** shared the same molecular formula with Compound **3** (C_9_H_18_N_2_O_3_) with two unsaturation degrees. The NMR data of **5** resemble those of **3** and **4**. Nevertheless, instead of two carbonyl carbons in **3** and **4**, the ^13^C-NMR showed resonances for one carbonyl carbon and signals for a methine carbon (δ_H_ 4.39, δ_C_ 85.4). Compound **5** must be monocyclic due to the lack of a second double bond, which is required for the unsaturation degrees implied by the molecular formula. Furthermore, the significant up field shift for the ^13^C resonance of the methine 1′-C (δ_C_ 85.4) suggested that 1′-C was connected to two nitrogen atoms. COSY and HMBC data allowed the construction of the structure as a monocyclic compound (4-hydroxy-3,5-diisopropyl-1,2,4-oxadiazinan-6-one) as shown in Figure 2. HRMS spectrum of **5** lacks the fragment peaks at *m/z* 158.1180 and 186.1129, which were observed for **3** and **4**, and showed instead a peak at *m/z* 170.1180 (C_9_H_16_NO_2_), which was assigned to the loss of NH_2_OH (Figure 3) further confirmed the structure of **5**. Based on the comparison of chemical structures, we postulate that **5** is a cyclization product of **4**, so **4** could be an intermediate in the biosynthesis of **5**. 

In order to evaluate the biological activity of Compounds **1**–**5**, they were tested for their antimicrobial, cytotoxic, anti-biofilm, and nematicidal activities, but none of them displayed any activity, even at the highest tested concentration of 300, 37, 33.33, and 100 μg/mL for those assays, respectively. Notably, Compound **4** did not possess any significant cytotoxicity against the tested human cervical carcinoma cell lines, although it has the same functional group as hydroxamic acid derivatives, which have been proposed by Pal and Saha [8] as powerful anticancer molecules. Moreover, it has also been found that pyrazine and a 2-naphthol bearing glucoside have lost the activity even though pyrazine and its analogues have been reported for decades to possess broad spectrum biological activities [9,10,11,12]. Even 2-naphthol has been proved to be an efficient moiety for the design of antimicrobial agents [13,14]. 

**Figure 4:**
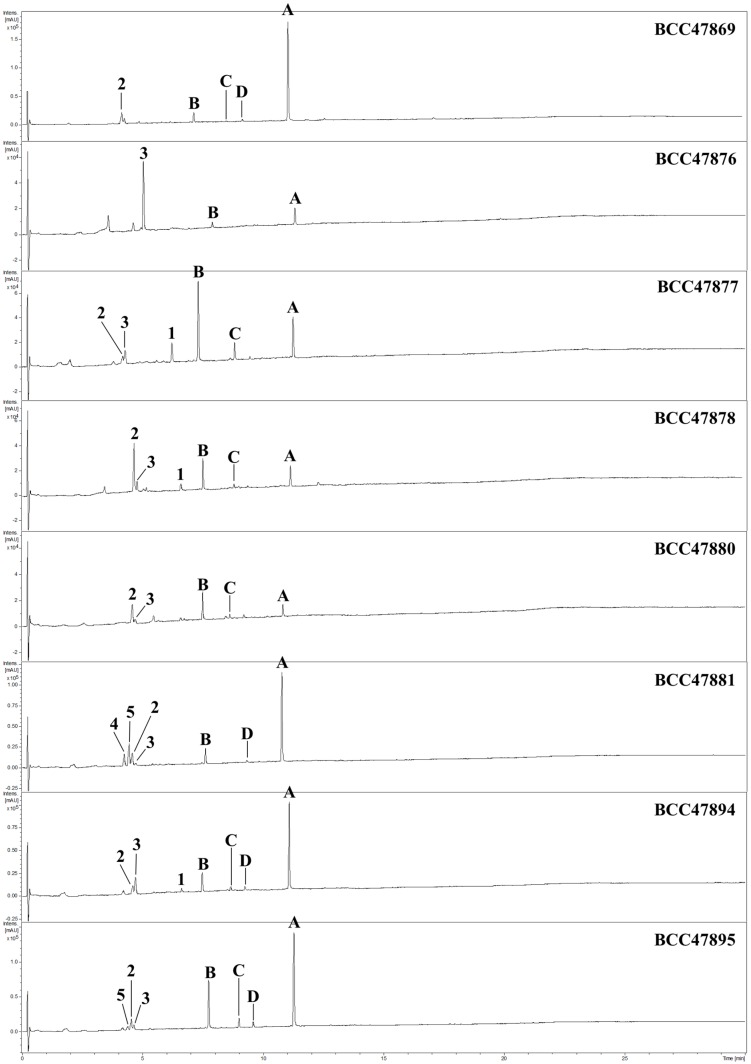
HPLC-UV profiles (200–600 nm) of *A. novoguineensis* isolates. Akanthol (**1**), akanthozine (**2**), Compound **3** (**3**), Compound **4** (**4**), Compound **5** (**5**), akanthopyrone A (**A**), akanthopyrone B (**B**), akanthopyrone C (**C**), and akanthopyrone D (**D**).

Due to further fieldwork, six more isolates of *A. novoguineensis* were encountered. Their HPLC profiles were generated and then compared with each other in order to study the distribution pattern of secondary metabolites in the species. Our concurrently reported akanthopyrones A and B were detected in all isolates in which half of them could produce akanthopyrone A as major metabolite (Figure 4). Most of the crude extracts contained Compounds **2** and **3** in low amounts except the strains BCC47878 and BCC47876, which showed great ability to produce those compounds as major components. Compounds **4** and **5** could not be found in any other isolates except BCC47895. Although the HPLC-UV profiles revealed that this isolate tended to produce trace amounts of compound **5**, the mass spectra did not exactly match. So far, there have been many reports on the secondary metabolites with structures related to the compounds produced by *A. novoguineensis*, but it is remarkable that those bearing the sugar moiety similar to akanthopyrones, akanthol, and akanthozine, were mostly isolated from scale insect pathogenic fungi, which belong to the order Hypocreales as well as *A. novoguineensis* [15,16,17,18,19,20,21].

## 3. Materials and Methods

### 3.1. General

1D and 2D NMR spectra were recorded on a Bruker Avance III 700 spectrometer with a 5 mm TXI cryoprobe (^1^H 700 MHz, ^13^C 175 MHz) and a Bruker Avance III 500 (^1^H 500 MHz, ^13^C 125 MHz) spectrometer; optical rotations were measured on a Perkin-Elmer 241 polarimeter, IR spectra were measured with a Nicolet Spectrum 100 FTIR spectrometer (Perkin-Elmer, Waltham, MA, USA). All HPLC-MS analyses were performed on Agilent 1260 Infinity Systems with a diode array detector and C_18_ Acquity UPLC BEH column (2.1 × 50 mm, 1.7 μm) from Waters with the gradient described by Helaly et al. [22] combined with ion trap MS (amazon speed, Bruker, Bremen, Germany), and HR-ESIMS spectra on a time-of-flight (TOF) MS (Maxis, Bruker). Chemicals and solvents were obtained from AppliChem GmbH (Darmstadt, Germany), Avantor Performance Materials (Deventor, The Netherlands), Carl Roth GmbH & Co. KG (Karlsruhe, Germany), and Merck KGaA (Darmstadt, Germany) in analytical and HPLC grade.

### 3.2. Fungal Material

Eight specimens of *A. novoguineensis* were collected from Ton-Nga-Chang Wildlife Sanctuary, Thailand. The fungal cultures were deposited in BIOTEC Culture Collection (BCC) as BCC47869, BCC47876, BCC47877, BCC47878, BCC47880, BCC47881, BCC47894, and BCC47895 and their 5.8S/ITS nrDNA were sequenced following the protocol described by Luangsa-ard et al. [23] and submitted to GenBank with accession number JX192676, JX192681, JX192682, JX192683, JX192684, JX192685, JX192691, and JX192692, respectively. The species description is provided in the supplementary data.

### 3.3. Fermentation and Extraction

Twenty mycelial plugs (0.5 × 0.5 cm^2^) were cut from actively growing colonies maintained on potato dextrose agar (PDA) and inoculated into 30 × 500 mL Erlenmeyer flask containing 150 mL of potato dextrose broth (PDB) supplemented with 0.1% of yeast extract. After incubation at room temperature (RT) under static condition for ten weeks, the culture filtrate was recovered by vacuum filtration and subsequently extracted according to the procedure described by Phainuphong et al. [24].

### 3.4. Isolation of Compounds ***1***–***5***

After the fermented broth of *A. novoguineensis* BCC47894 was extracted with 4 L of ethyl acetate (EtOAc), and the solvent was evaporated to dryness. The oily residue was then dissolved in methanol and further fractionated using an Agilent 1100 series HPLC system (Agilent Technologies, Wilmington, DE, USA). A reverse-phase C18 column (Kromasil 250 × 20 mm, 7 μm; MZ analysentechnik, Mainz, Germany) was used as a stationary phase and a mixture of deionized water (Milli-Q, Millipore, Schwalbach, Germany, solvent A) and acetonitrile (ACN, HPLC-grade, solvent B) were used as mobile phase. The separation was carried out according to the following gradient: linear from 20 to 80% solvent B in 30 min, afterwards linear gradient to 100% solvent B in 5 min, thereafter isocratic conditions at 100% for 5 min, with a flow rate of 20 mL/min. UV detection was carried out at 210, 280, and 354 nm, and fractions were collected and combined according to the observed peaks. Compound **1** (1.4 mg) was obtained at a retention time (*t*_R_) = 11–12 min, Compound **2** (2.6 mg) at *t*_R_ = 5–6 min, and Compound **3** (11.3 mg) at *t*_R_ = 7–8 min.

The EtOAc extract from culture filtrate of the fungus BCC47881 dissolved in methanol was subjected to preparative HPLC using the system described above to provide Compound **5** (4.6 mg) at *t*_R_ = 9–10 min. The fraction obtained at a retention time of 6–7 min was repeatedly fractionated using a reverse-phase C18 column (Kromasil, 250 × 20 mm, 7 μm; flow rate 20 mL/min), applying a linear gradient from 5 to 30% solvent B for the first 30 min, increasing to 100% for over 5 min, followed by an isocratic condition at 100% solvent B for 5 min. Compound **4** (13.69 mg) eluted at 20–21 min.

***Akanthol* (1)**: brown gum; ${\left[\alpha \right]}_{D}^{25}$ +10° (*c* 0.1, MeOH); ^1^H- and ^13^C-NMR in DMSO-*d*_6_ see Table 1. ^1^H-NMR (700 MHz, METHANOL-*d*_4_): δ_H_ 7.51 (8-H, *dd*, *J* = 1.9 and 7.5 Hz), 7.33 (7-H, *dd*, *J* = 7.5 and 7.3 Hz), 7.31 (4-H, *dd*, overlapping), 7.30 (1-H, *s*), 7.31 (6-H, *d*, overlapping), 6.81 (3-H, *t*, *J* = 4.3 Hz), 5.09 (1′-H, *d*, *J* = 7.7 Hz), 3.91 (6′a-H, *dd*, *J* = 2.2 and 12.5 Hz), 3.76 (6′b-H, *dd*, *J* = 4.9 and 12.3 Hz), 3.61 (OMe, *s*), 3.60 (3′-H, *dd*, *J* = 8.6 and 9.5 Hz), 3.57 (2′-H, *dd*, *J* = 7.7 and 9.5 Hz), 3.51 (5′-H, *ddd*, *J* = 2.2, 4.7 and 9.7 Hz), 3.26 (4′-H, *dd*, *J* = 8.6 and 9.7 Hz). δ_C_ (175 MHz, METHANOL-*d*_4_): 155.9 (C-2), 155.2 (C-5), 138.5 (C-8a), 128.5 (C-7), 127.1 (C-4), 124.7 (C-8), 120.3 (C-1), 117.0 (C-4a), 111.8 (C-6), 111.9 (C-3), 104.4 (C-1′), 80.7 (C-4′), 78.4 (C-3′), 77.9 (C-5′), 75.3 (C-2′), 62.2 (C-6′), 61.1 (OMe). Sugar signals appeared less overlapping in the ^1^H-NMR spectrum in methanol-*d*_4_, while the aromatic signals were less overlapping in the ^1^H-NMR spectrum measured in DMSO-*d*_6_. LCMS *m/z* 337 [M + H]^+^ (17), 359 [M + Na]^+^ (73), 695 [2M + Na]^+^ (100), 161 [M + H-4-*O*-methyl-glucopyranose]^+^ (26), 435 [M − H]^−^ (100), 159 [M − H-4-*O*-methyl-glucopyranose]^−^ (84); HRESIMS *m/z* 359.1107 [M + Na]^+^ (calcd. for C_17_H_20_O_7_Na^+^, 359.1101). Spectra see Supplementary Material.

***Akanthozine* (2)**: brown gum; ${\left[\alpha \right]}_{D}^{25}$ +20° (*c* 0.17, MeOH); ^1^H- and ^13^C-NMR see Table 1. LCMS *m*/*z* 373 [M + H]^+^ (84), 359 [M + Na]^+^ (48), 197 [M + H-4-O-methyl-glucopyranose]^+^ (100), 371 [M − H]^−^ (100), 743 [2M − H]^−^ (5); HRESIMS *m/z* 373.1971 [M + H]^+^ (calcd. for C_17_H_29_N_2_O_7_^+^, 373.1969). Spectra see Supplementary Material.

***Compound* 3**: white amorphous powder; ${\left[\alpha \right]}_{D}^{25}$ +6° (*c* 0.99, MeOH); ^1^H-NMR (500 MHz, METHANOL-*d*_4_): δ_H_ 4.61 (2-H, *d*, *J* = 10.1 Hz), 3.22 (2′-H, *m*), 2.38 (3-H, *m*), 1.13 (4′-H, *d*, *J* = 7.0 Hz), 1.11 (3′-H, *d*, *J* = 6.7 Hz), 1.02 (4-H, *d*, *J* = 6.7 Hz), 0.96 (5-H, *d*, *J* = 6.4 Hz). δ_C_ (125 MHz, METHANOL-*d*_4_): 180.6 (C-1′), 175.1 (C-1), 65.8 (C-2), 31.3 (C-2′), 28.4 (C-3), 19.8 (C-4), 19.8 (C-5) 19.5 (C-3′), 19.1 (C-4′). ^15^N-NMR (700 MHz, METHANOL-*d*_4_): δ_N_ 106.4 (NH_2_), 177.2 (ter. N). IR (KBr) υ 3346, 3319, 2943, 2831, 1448, 1366, 1113, 1022 cm^−1^, LCMS *m/z* 203 [M + H]^+^ (4), 225 [M + Na]^+^ (33), 201 [M − H]^−^ (26); HRESIMS *m/z* 203.1389 [M + H]^+^ (calcd. for C_9_H_19_N_2_O_3_^+^, 203.1390). Spectra see Supplementary Material.

***Compound* 4**: white amorphous powder; ${\left[\alpha \right]}_{D}^{25}$ 53° (*c* 1.7, MeOH); ^1^H-NMR (500 MHz, METHANOL-*d*_4_): δ_H_ 4.46 (2-H, *d*, *J* = 10.7 Hz), 3.21 (2′-H, *m*), 2.45 (3-H, *td*, *J* = 6.6 and 10.5 Hz), 1.11 (4′-H, *d*, *J* = 6.7 Hz), 1.09 (3′-H, *d*, *J* = 7.0 Hz), 0.96 (4-H, *d*, *J* = 6.7 Hz), 0.92 (5-H, *d*, *J* = 6.4 Hz). δ_C_ (125 MHz, METHANOL-*d*_4_): 180.4 (C-1′), 169.1 (C-1), 63.6 (C-2), 31.3 (C-2′), 28.0 (C-3), 19.9 (C-4′), 19.8 (C-4), 19.7 (C-5), 19.4 (C-3′). IR (KBr) υ 3466, 3326, 2944, 2831, 1738, 1448, 1366, 1217, 1022 cm^−1^, LCMS *m/z* 241 [M + Na]^+^ (20), 219 [M + H]^+^ (3), 217 [M − H]^−^ (100); HRESIMS *m/z* 219.1343 [M + H]^+^ (calcd. for C_9_H_19_N_2_O_4_^+^, 219.1339). Spectra see Supplementary Material.

***Compound* 5**: pale brown gum; ${\left[\alpha \right]}_{D}^{25}$ +12° (*c* 0.3, MeOH); ^1^H-NMR (500 MHz, CHLOROFORM-*d*): δ_H_ 4.39 (1´-H, *d*, *J* = 2.4 Hz), 3.47 (2-H, *d*, *J* = 3.7 Hz), 2.26 (2´-H, *m*), 2.13 (3-H, *m*), 1.08 (4´-H, *d*, *J* = 7.0 Hz), 1.05 (4-H, *d*, *J* = 7.0 Hz), 0.98 (3´-H, *d*, *J* = 7.0 Hz), 0.97 (5-H, *d*, *J* = 7.0 Hz). δ_C_ (125 MHz, CHLOROFORM-d): 167.0 (C-1), 85.7 (C-1´), 72.6 (C-2), 29.3 (C-3), 29.2 (C-2´), 18.9 (C-4), 17.8 (C-5), 17.4 (C-4´), 15.9 (C-3´). LCMS *m/z* 203 [M + H]^+^ (7), 427 [2M + Na]^+^ (9), 201 [M − H]^−^ (30), 403 [2M − H]^−^; HRESIMS *m/z* 203.1395 [M + H]^+^ (calcd. for C_9_H_19_N_2_O_3_^+^, 203.1390). Spectra see Supplementary Material.

### 3.5. Acid Hydrolysis of Akanthol *(**1**)*

Compound **1** (1 mg) was hydrolyzed with 10% aqueous HCl (1 mL) at 90 °C for 12 h. The reaction mixture was then diluted with H_2_O (2 mL) and extracted with EtOAc (2 × 3 mL). The aqueous layer was concentrated under vacuum to yield 4-*O*-methyl-d-glycopyranose (${\left[\alpha \right]}_{D}^{25}$ +36, *c* 0.05, MeOH).

### 3.6. Biological Activities

#### 3.6.1. Antimicrobial Activity and Cytotoxicity Assay

All isolated compounds were determined for the minimum inhibitory concentration (MIC) against *Bacillus subtilis* DSM10, *Escherichia coli* DSM498, *Candida tenuis* MUCL29892, and *Mucor plumbeus* MUCL49355, and the cytotoxicity against the cervix carcinoma cell line KB-3-1 and the established mouse fibroblast cell line L929 using the protocols previously described by Richter et al. [25].

#### 3.6.2. Anti-Biofilm Activity Assay

The determination of the ability of new compounds to prevent biofilm formation of *Staphylococcus aureus* DSM1104 (ATCC25923) and *Pseudomonas aeruginosa* PA14 [26] was performed using the microtiter dish biofilm formation assay described by O’Toole [27] with minor modifications. The biofilm forming strains were enriched overnight in CASO medium with and without 4% glucose for *S. aureus* and *P. aeruginosa*, respectively. After incubation at 37 °C under shaking condition, the overnight cultures were adjusted to equal the turbidity of 0.5 McFarland standard using medium. The assay was carried out in 96-well tissue cultured-treated microplates (TPP^®^, Trasadingen, Switzerland) for *S. aureus* and non-treated plates (Falcon^®^MicroTest™, Albany, NY, USA) for *P. aeruginosa*, in which 10 μL of each compound (0.5 mg/mL) in six replicates were mixed together with 140 μL of bacterial suspension. Methanol and CASO medium with and without 4% glucose were used as a negative control and tetracycline (100 μg/mL) as a positive control. Plates were covered with a sterile adhesive porous paper (Kisker Biotech GmbH, Steinfurt, Germany) and incubated at 37 °C for 24 h. After incubation, bacterial biofilms were stained with 0.1% crystal violet solution (Fluka, Steinheim, Germany) following the protocol of O’Toole [27].

#### 3.6.3. Nematicidal Activity Assay

The nematicidal activity of the new compounds against *Caenorhabditis elegans* was tested in a microwell plate assay slightly modified from the method reported by Stadler et al. [28]. The compounds were dissolved in methanol to a concentration of 100 mg/mL and subsequently diluted 1:100 with sterile distilled water. The free-living nematode, *C. elegans* was monoxenically cultured on nematodes agar (soy peptone 2 g, NaCl 1 g, Agar 20 g, 1000 mL of distilled water, after autoclaving, the following ingredients were added: cholesterol (1 mg/mL EtOH), 0.5 mL, 1 M CaCl_2_, 1 mL, 1 M MgSO_4_, 1 mL, 40 mM potassium phosphate buffer, 12.5 mL, pH 6.8) with living *E. coli* DSM498, at 20 °C in the dark for a week. After incubation, adult nematodes were suspended in sterile distilled water and transferred to a sterile tube. Finally, the nematodes were counted using a Malassez counting chamber and further adjusted to obtain a concentration of 500 nematodes/mL with sterile distilled water. Nematicidal activity screening was performed in a 24-microwell plate (total volume 1 mL/well) and 4 concentrations of 100, 50, 20, and 10 μg/mL of each compound were tested. Standard nematicide, ivermectin, and 1% MeOH were used as positive inhibitory control and solvent control, respectively. The plate was incubated at 20 °C in the dark and nematicidal activity was recorded after 18 h of incubation.

## 4. Conclusions

Two new natural products bearing 4-*O*-methyl-β-d-glucopyranose, akanthol (**1**), akanthozine (**2**), and three new amide derivatives including a hydroxamic acid derivative (**3**–**5**) were obtained from the spider-associated fungus *Akanthomyces novoguineensis* and proved to be the species-specific secondary metabolites in addition to the recent reported akanthopyrones A–D. The new metabolites showed no significant activities in antimicrobial, cytotoxic, anti-biofilm, and nematicidal assays. 

## Figures and Tables

**Table 1:** ^1^H- and ^13^C-NMR data for akanthol (**1**) in (500 MHz, DMSO-*d*_6_) and akanthozine (**2**) (500 MHz, Methanol-*d*_4_).

Pos	1		Pos	2	
	δ_H_ (*J* in Hz)	δ_C_, Type		δ_H_ (*J* in Hz)	δ_C_, Type
1	7.35, s	118.8, CH	2	-	154.9, C
2	-	153.5, C	3	-	139.5, C
3	6.81, *dd*, 3.4, 5.2	110.6, CH	5	-	147.6, C
4	7.35, *dd*, 6.5, 3.4	127.4, CH	6	-	144.7, C
4a	-	115.1, C	7	3.24, *m*	30.6, CH
5	-	154.1, C	8	1.20, *d*, 3.7	21.1, CH_3_
6	7.30, *brdd*, 0.9, 7.7	110.5, CH	9	1.19, *d*, 3.4	21.0, CH_3_
7	7.37, *dd*,8.0, 7.7	126.2, CH	10	3.31, *m*	28.7, CH
8	7.55, *dd*, 0.9, 8.2	123.0, CH	11	1.23, *d*, 4.0	21.2, CH_3_
8a	-	136.3, C	12	1.22, *d*, 4.6	21.2, CH_3_
OH	9.29	-			
4-*O*-methyl-β-d-glucopyranose					
1′	5.07, *d*, 8.2	102.5, CH	1′	5.63, *d*, 7.9	98.8, CH
2′	3.40, *dd*, 8.2, 8.6	73.7, CH	2′	3.51, *dd*, 8.1, 8.2	74.9, CH
3′	3.48, overlapping	76.0, CH	3′	3.58, *dd*, 8.9, 9.5	78.9, CH
4′	3.10, *dd*, 9.0, 9.5	79.1, CH	4′	3.21, *dd*, 8.9, 8.2	80.8, CH
5′	3.50, overlapping	76.2, CH	5′	3.32, *m*	77.7, CH
6′	3.72, *ddd*, 11.9, 4.8, 1.7	60.3, CH_2_	6′	3.76, *dd*, 2.1, 12.2	62.2, CH_2_
	3.56, *dd*, 11.6, 5.1			3.67, *dd*, 4.4, 12.1	
OMe	3.48, *s*	59.7, CH_3_	OMe	3.59, *s*	61.0, CH_3_

## Supplementary Materials

Supplementary materials are available online. The species description of the producers, UV, HRESIMS and ^1^H, ^13^C, COSY, HSQC, HMBC NMR spectra of Compounds **1**–**5** are available as Supplementary Material.
