# Supplementary material for: Five Unprecedented Secondary Metabolites from the Spider Parasitic Fungus Akanthomyces novoguineensis
Source: Molecules. 2017 Jun 14;22(6):991. doi: 10.3390/molecules22060991 (PMC6152716; doi:10.3390/molecules22060991)
Supplement: Supplementary file 1 [file molecules-22-00991-s001.pdf]

Supplementary Materials

Five new secondary metabolites; akanthol, akanthozine and three amide derivatives from the spiderparasitic fungus *Akanthomyces novoguineensis*

Soleiman E. Helaly[1,2,a], Wilawan Kuephadungphan[1,3,a], Souwalak Phongpaichit[3,4], Janet Jennifer Luangsa-ard[5], Vatcharin Rukachaisirikul[6,7], Marc Stadler[1,*]

Affiliation

[1] Department of Microbial Drugs, Helmholtz Centre for Infection Research, Braunschweig 38124, Germany

[2] Department of Chemistry, Faculty of Science, Aswan University, Aswan 81528, Egypt

[3] Department of Microbiology, Faculty of Science, Prince of Songkla University, Songkhla 90112, Thailand

[4] Natural Products Research Center of Excellence and Department of Microbiology, Prince of Songkla University, Songkhla 90112, Thailand

[5] National Centre for Genetic Engineering and Biotechnology (BIOTEC), Pathumthani 12120, Thailand

[6] Department of Chemistry, Faculty of Science, Prince of Songkla University, Songkhla 90112, Thailand

[7] Center of Excellence for Innovation in Chemistry, Prince of Songkla University, Songkhla 90112, Thailand

Correspondence

Prof. Dr. Marc Stadler
Department of Microbial Drugs, Helmholtz Centre for Infection Research, Inhoffenstrasse 7, 38124 Braunschweig, Germany
E-mail: marc.stadler@helmholtz-hzi.de
Phone: +49 531 6181-4240
Fax: +49 531 6181-9499

[a] These authors contributed equally to this work

Isolation and characterization of fungal materials

The isolation of pure cultures was performed immediately after the fungal specimens were collected. Briefly, agar plug of potato dextrose agar (PDA) containing 50 mg/L penicillin and streptomycin were cut into small pieces and then gently swiped over the spores located on synnemata using a fine sterile needle. Agar plugs with spores were placed on PDA containing antibiotics which was then incubated at 25 ºC and the conidial germination was observed daily as well as fungal contamination. Pure cultures were isolated onto fresh PDA plate without antibiotics by hyphal tip isolation, allowed to grow for 4-6 weeks and subsequently deposited to Prince of Songkla University and BIOTEC, Thailand with the BCC code no. BCC47869 (EPF036), BCC47876 (EPF057), BCC47877 (EPF063), BCC47878 (EPF068), BCC47880 (EPF070), BCC47881 (EPF071), BCC47894 (EPF097) and BCC47895 (EPF098).

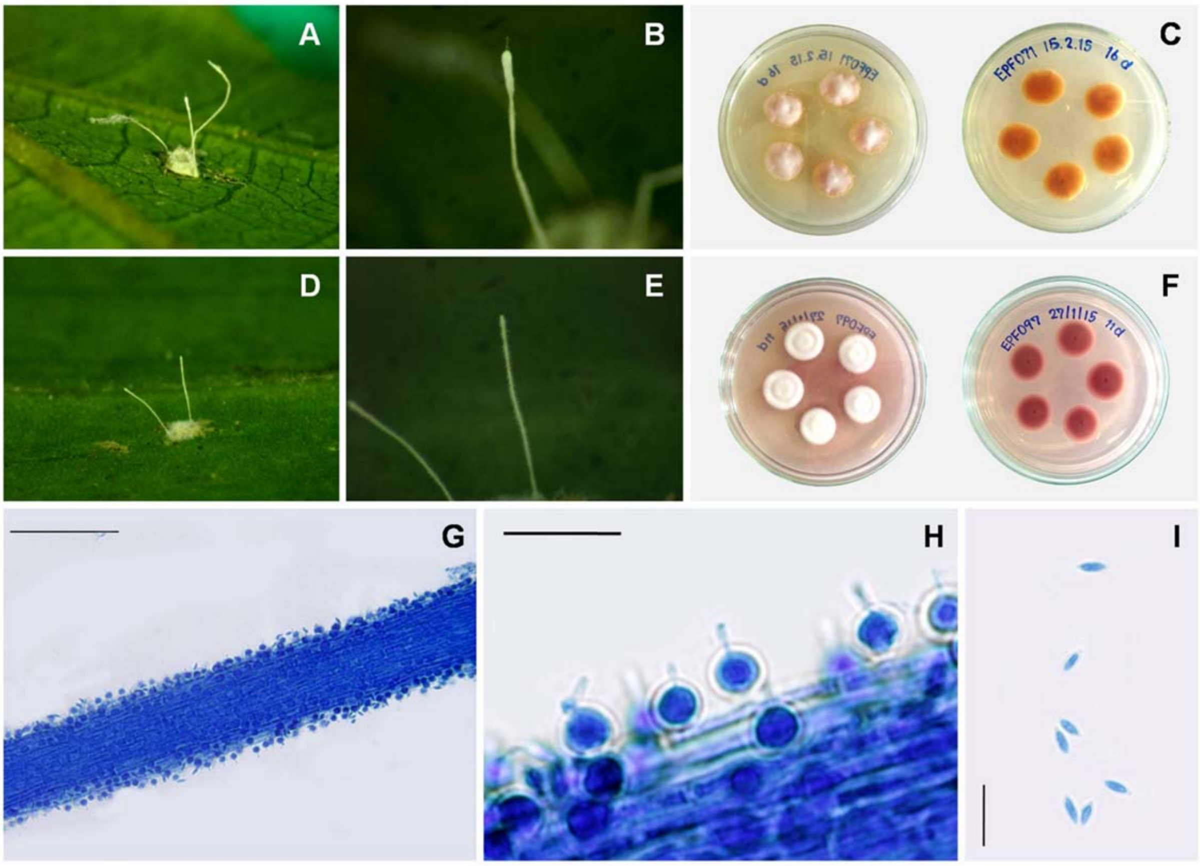

Figure S1. Morphological characteristics of *A. novoguineensis*. *A. novoguineensis* BCC47881 and its colonies on PDA at 25 ºC for 16 days (A-C), BCC47894 and its colonies on PDA at 25 ºC for 11 days (D-F), part of synnema showing phialides (G-H), conidia (I), Scale bar: G = 500 µm, H-I = 10 µm

The producing strains were characterized as *Akanthomyces novoguineensis* according to Samson and Brady [1], Hywel-Jones [2] and Hsieh et al. [3] by producing 2-5 creamish white, erect, cylindrical synnemata on the hosts (Figure S1). The globose, hyaline, smooth-walled phialides, each with a single

distinct neck were located scatteringly along the entire length of synnema (Figure S1). The conidia forming at the tip of the neck were hyaline and ellipsoid (Figure S1). On PDA, the white to cream colonies were slow-growing, reaching a diameter of 0.8-1.0 cm in 14 days at 25 ºC. The colony reverse was pink or yellowish-orange with diffused pale pink or yellow pigment in the agar (Figure S1). Sporulation was not found.

Based on the nucleotide-nucleotide search using the Basic Local Alignment Search Tool (BLAST) from the National Center for Biotechnology Information (NCBI, USA), the ITS rDNA sequences of the producers were most closely related to *A. novoguineensis*and the strain BCC41865 (GenBank accession no. JN201872) was the top BLAST match with 99% identity for all isolates except BCC47894 (97% identity).

According to morphological features and the BLAST search results, the species could be identified as *A. novoguineensis*.

Table S1. A list of species of Cordycipitaceae in which akanthopyrones [4] and compounds **1-5** were not detected in their EtOAc extracts (4.5 mg/mL) of fermentation broths using HPLC profiling. The fungal isolates were cultured in YMG medium (yeast extract, 4 g; malt extract, 10 g; D-glucose, 4 g; distilled water, 1,000 mL) or PDB supplemented with 0.1% of yeast extract or both.

**Original code**	**Species**	**Medium**
NHJ06753	*Akanthomyces arachnophilus* (Petch) Samson & H.C. Evans	YMG
NHJ11602	*A. arachnophilus* (Petch) Samson & H.C. Evans	YMG
NHJ11619	*A. arachnophilus* (Petch) Samson & H.C. Evans	YMG
EPF094	*A. cinereus* Hywel-Jones	YMG, PDB
EPF128	*A. cinereus* Hywel-Jones	YMG, PDB
EPF155	*A. cinereus* Hywel-Jones	YMG
EPF156	*A. cinereus* Hywel-Jones	YMG
MY04459	*A. cinereus* Hywel-Jones	YMG, PDB
MY04461	*A. cinereus* Hywel-Jones	YMG, PDB
NHJ05984	*A. cinereus* Hywel-Jones	YMG
EPF069	*A. novoguineensis* Samson & B.L. Brady	YMG
EPF071	*A. novoguineensis* Samson & B.L. Brady	YMG
EPF093	*A. novoguineensis* Samson & B.L. Brady	YMG
EPF098	*A. novoguineensis* Samson & B.L. Brady	YMG
EPF141	*A. novoguineensis* Samson & B.L. Brady	YMG
MY01626	*A. novoguineensis* Samson & B.L. Brady	YMG
MY01627	*A. novoguineensis* Samson & B.L. Brady	YMG
MY02831	*A. novoguineensis* Samson & B.L. Brady	YMG
MY00736	*A. pistillariiformis* (Pat.) Samson & H.C. Evans	YMG
MY00830	*A. pistillariiformis* (Pat.) Samson & H.C. Evans	YMG
MY00832	*A. pistillariiformis* (Pat.) Samson & H.C. Evans	YMG
MY04489	*A. websteri* Hywel-Jones	YMG
MY06006.02	*Akanthomyces* sp.	YMG

Table S1 (Cont.) A list of species of Cordycipitaceae in which akanthopyrones [4] and compounds **1-5** were not detected in their EtOAc extracts (4.5 mg/mL) of fermentation broths using HPLC profiling. The fungal isolates were cultured in YMG medium (yeast extract, 4 g; malt extract, 10 g; D-glucose, 4 g; distilled water, 1,000 mL) or PDB supplemented with 0.1% of yeast extract or both.

**Original code**	**Species**	**Medium**
MY04258	*Cordyceps nelumboides* Kobayasi & Shimizu	YMG
MY06110	*C. nelumboides* Kobayasi & Shimizu	YMG
MY01458.02	*C. tuberculata* (Lebert) Maire	YMG
MY01459	*C. tuberculata* (Lebert) Maire	YMG
EPF083	*Gibellula pulchra* Cavara	YMG
MY02556	*G. leiopus* (Vuill. Ex Maubl.) Mains	YMG
MY02561	*G. leiopus* (Vuill. Ex Maubl.) Mains	YMG
NHJ12168-2	*G. leiopus* (Vuill. Ex Maubl.) Mains	YMG
EPF004	*Gibellula* sp.	YMG
EPF034	*Gibellula* sp.	YMG
EPF060	*Gibellula* sp.	PDB
EPF079	*Gibellula* sp.	PDB
EPF081	*Gibellula* sp.	PDB
EPF120	*Gibellula* sp.	YMG
EPF147	*Gibellula* sp.	PDB
EPF150	*Gibellula* sp.	PDB
EPF169	*Gibellula* sp.	PDB
EPF171	*Gibellula* sp.	PDB
EPF172	*Gibellula* sp.	YMG, PDB
EPF173	*Gibellula* sp.	YMG, PDB
EPF174	*Gibellula* sp.	YMG, PDB
EPF175	*Gibellula* sp.	PDB
EPF180	*Gibellula* sp.	PDB
EPF182	*Gibellula* sp.	PDB
EPF183	*Gibellula* sp.	PDB
MY04487.02	*Gibellula* sp.	YMG
MY04894	*Gibellula* sp.	YMG
MY05051.02	*Gibellula* sp.	YMG
MY05247.02	*Gibellula* sp.	YMG
MY05842.02	*Gibellula* sp.	YMG
MY06077.02	*Gibellula* sp.	YMG
MY06216.02	*Gibellula* sp.	YMG
MY06357	*Gibellula* sp.	YMG
MY06585	*Gibellula* sp.	YMG
MY01654	*Isaria cateniannulata* (Z.Q. Liang) Samson & Hywel-Jones	YMG
NHJ05763	*I. cateniannulata* (Z.Q. Liang) Samson & Hywel-Jones	YMG
MY01338	*I. farinosa* (Holmsk.) Fr.	YMG
MY03945	*I. farinosa* (Holmsk.) Fr.	YMG
MY00700	*I. fumosorosea* Wize	YMG
MY01362	*I. javanica* (Bally) Samson & Hywel-Jones	YMG

Table S1 (Cont.) A list of species of Cordycipitaceae in which akanthopyrones [4] and compounds **1-5** were not detected in their EtOAc extracts (4.5 mg/mL) of fermentation broths using HPLC profiling. The fungal isolates were cultured in YMG medium (yeast extract, 4 g; malt extract, 10 g; D-glucose, 4 g; distilled water, 1,000 mL) or PDB supplemented with 0.1% of yeast extract or both.

**Original code**	**Species**	**Medium**
MY01820	*I. javanica* (Bally) Samson & Hywel-Jones	YMG
MY01821	*I. javanica* (Bally) Samson & Hywel-Jones	YMG
MY02252	*I. javanica* (Bally) Samson & Hywel-Jones	YMG
MY02849	*I. javanica* (Bally) Samson & Hywel-Jones	YMG
MY02853	*I. javanica* (Bally) Samson & Hywel-Jones	YMG
MY02946.01	*I. javanica* (Bally) Samson & Hywel-Jones	YMG
MY02948	*I. javanica* (Bally) Samson & Hywel-Jones	YMG
MY02949	*I. javanica* (Bally) Samson & Hywel-Jones	YMG
MY02951	*I. javanica* (Bally) Samson & Hywel-Jones	YMG
NHJ02458	*I. javanica* (Bally) Samson & Hywel-Jones	YMG
NHJ03004	*I. javanica* (Bally) Samson & Hywel-Jones	YMG
NHJ13336	*I. javanica* (Bally) Samson & Hywel-Jones	YMG
MY08816	*I. takamizusanensis* Kobayasi	YMG
MY08817	*I. takamizusanensis* Kobayasi	YMG
MY08819	*I. takamizusanensis* Kobayasi	YMG
MY08820	*I. takamizusanensis* Kobayasi	YMG
MY08821	*I. takamizusanensis* Kobayasi	YMG
MY01146	*I. tenuipes* Peck	YMG
MY03904	*I. tenuipes* Peck	YMG
MY03940	*I. tenuipes* Peck	YMG
MY00949	*Isaria* sp.	YMG
MY00954	*Isaria* sp.	YMG
MY01037	*Isaria* sp.	YMG
MY01787	*Isaria* sp.	YMG
MY01822	*Isaria* sp.	YMG
MY03195	*Isaria* sp.	YMG
MY04316	*Isaria* sp.	YMG
MY04954	*Isaria* sp.	YMG
MY06686.01	*Isaria* sp.	YMG
MY06686.02	*Isaria* sp.	YMG
MY05246.01	*Torrubiella* sp.	YMG
MY05247.01	*Torrubiella* sp.	YMG
MY05248.01	*Torrubiella* sp.	YMG
MY05255	*Torrubiella* sp.	YMG
MY05583.01	*Torrubiella* sp.	YMG
MY05979.01	*Torrubiella* sp.	YMG
MY06006.01	*Torrubiella* sp.	YMG
MY06077.01	*Torrubiella* sp.	YMG
MY06113.01	*Torrubiella* sp.	YMG
MY06216.01	*Torrubiella* sp.	YMG
MY06535.01	*Torrubiella* sp.	YMG
MY06567.01	*Torrubiella* sp.	YMG

References

1. Samson, R. A.; Brady, B. L. *Akanthomyces novoguineensis* sp. nov. *Trans. Br. Mycol. Soc.* **1982**, *79*, 571–572.

2. Hywel-Jones, N. *Akanthomyces* on spiders in Thailand. *Mycol. Res.* **1996**, *100*, 1065–1070.

3. Hsieh, L. S.; Tzean, S. S.; Wu, W. J. The genus *Akanthomyces* on spiders from Taiwan. *Mycol. Soc. Am.* **1997**, *89*, 319–324.

4. Kuephadungphan, W.; Helaly, S. E.; Daengrot, C.; Phongpaichit, S.; Luangsa-Ard, J. J.; Rukachaisirikul, V.; Stadler, M. Akanthopyrones A – D, α-pyrones bearing a 4-O-methyl-β-D-glucopyranose moiety from the spider-associated ascomycete *Akanthomyces novoguineensis*. *Phytochemistry* **2017** (submitted).

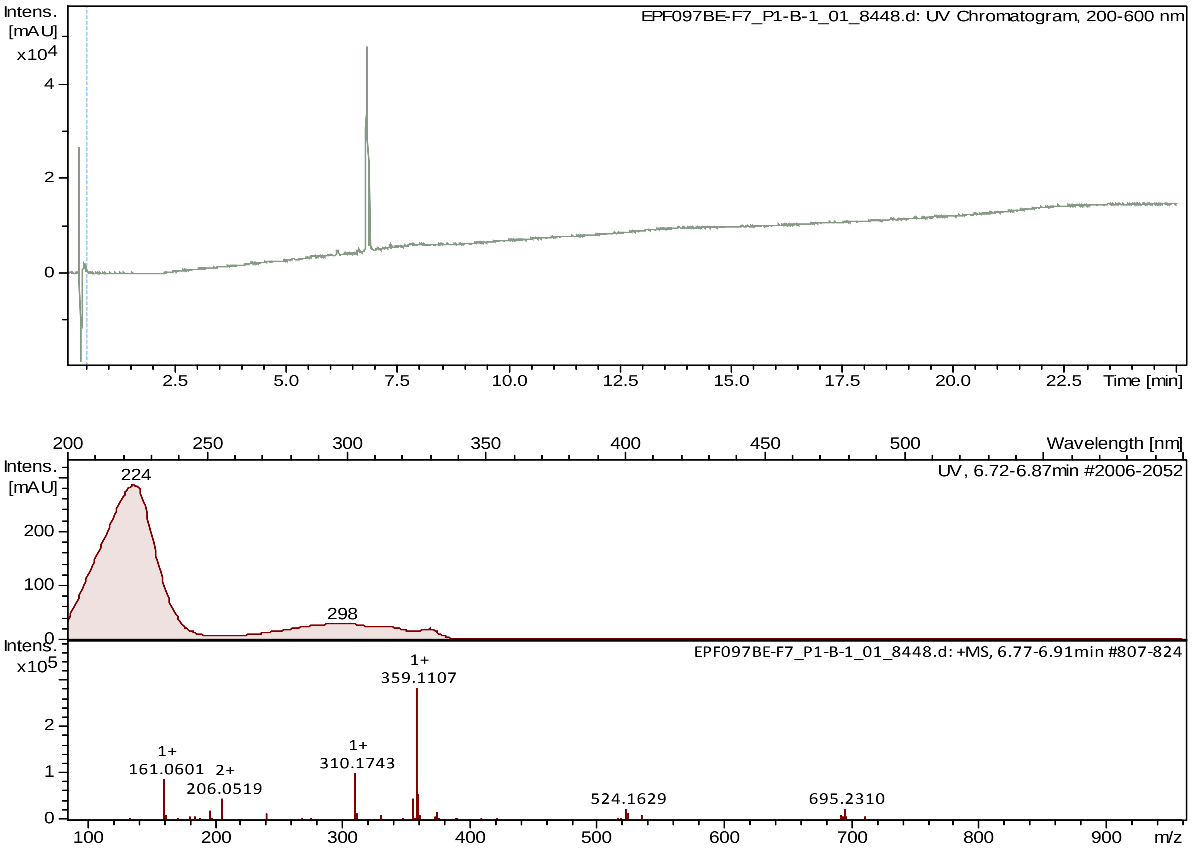

Figure S2. HR (+) ESIMS spectra of akanthol (**1**)

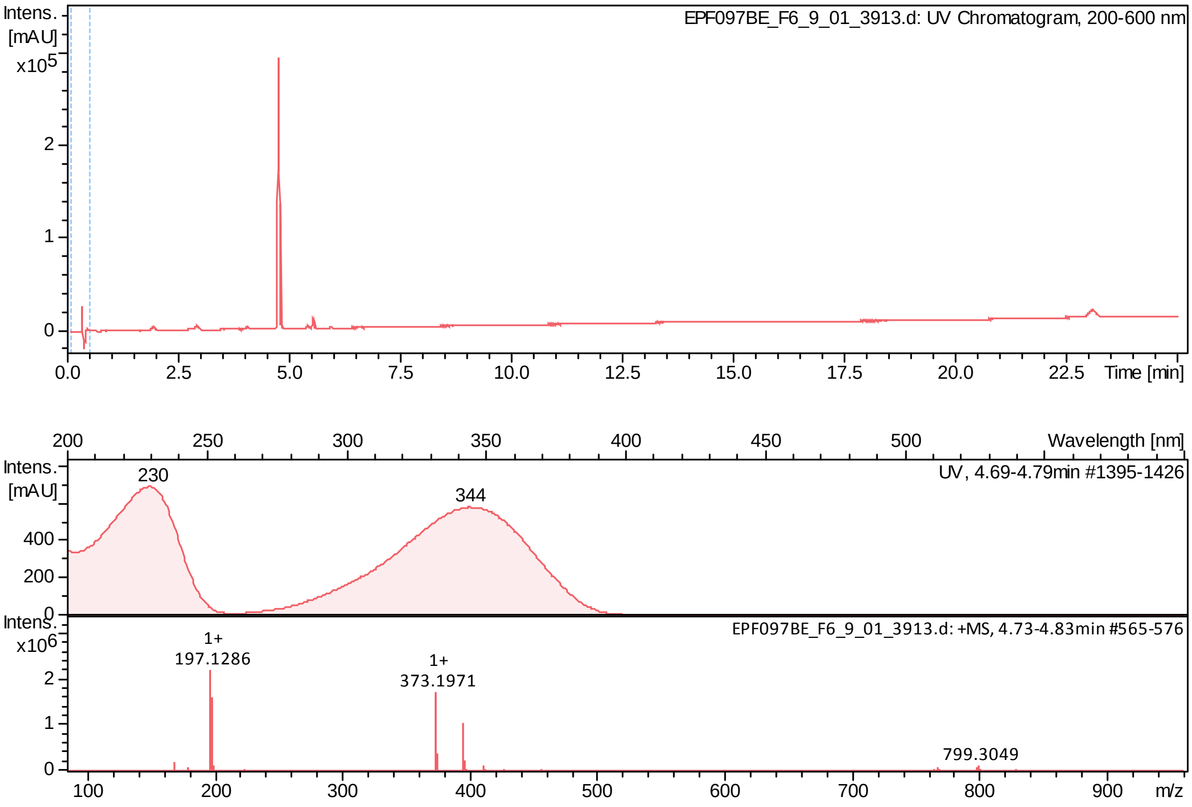

Figure S3. HR (+) ESIMS spectra of akanthozine (**2**)

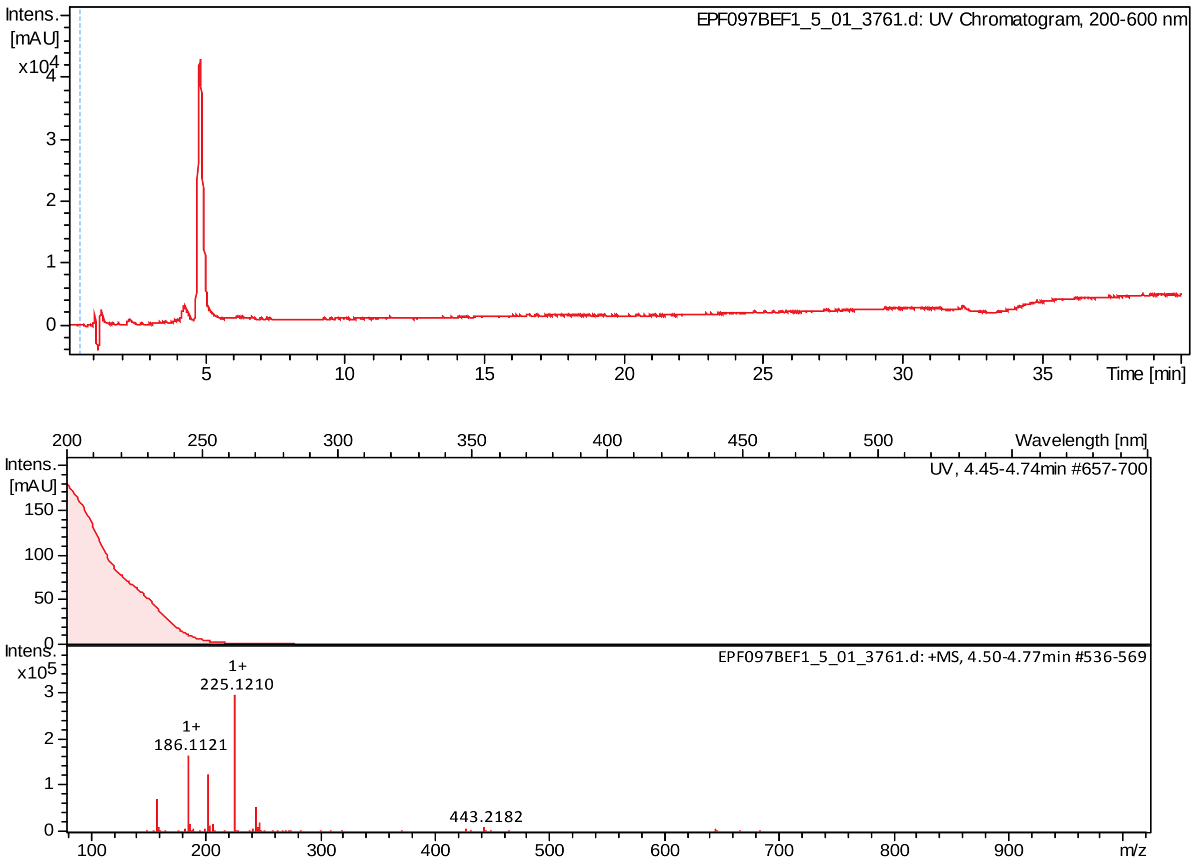

Figure S4. HR (+) ESIMS spectra of compound **3**

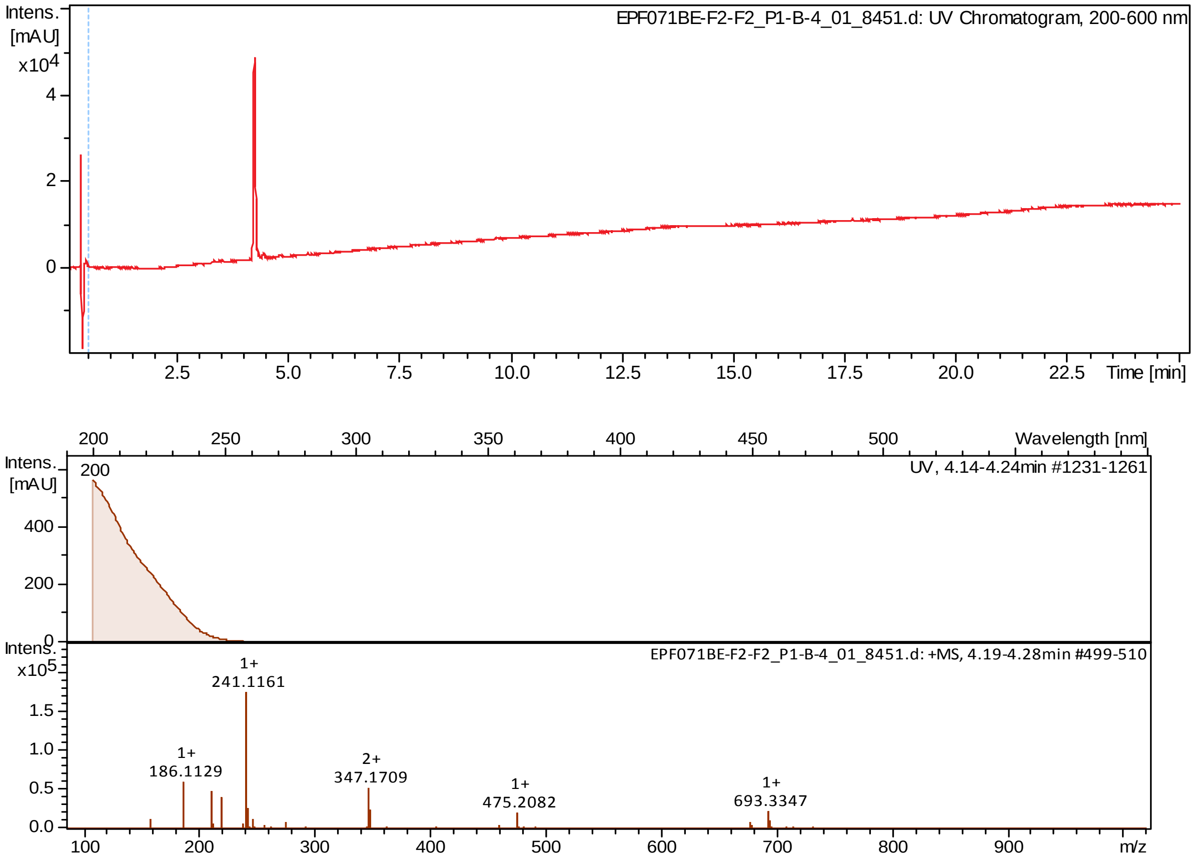

Figure S5. HR (+) ESIMS spectra of compound **4**

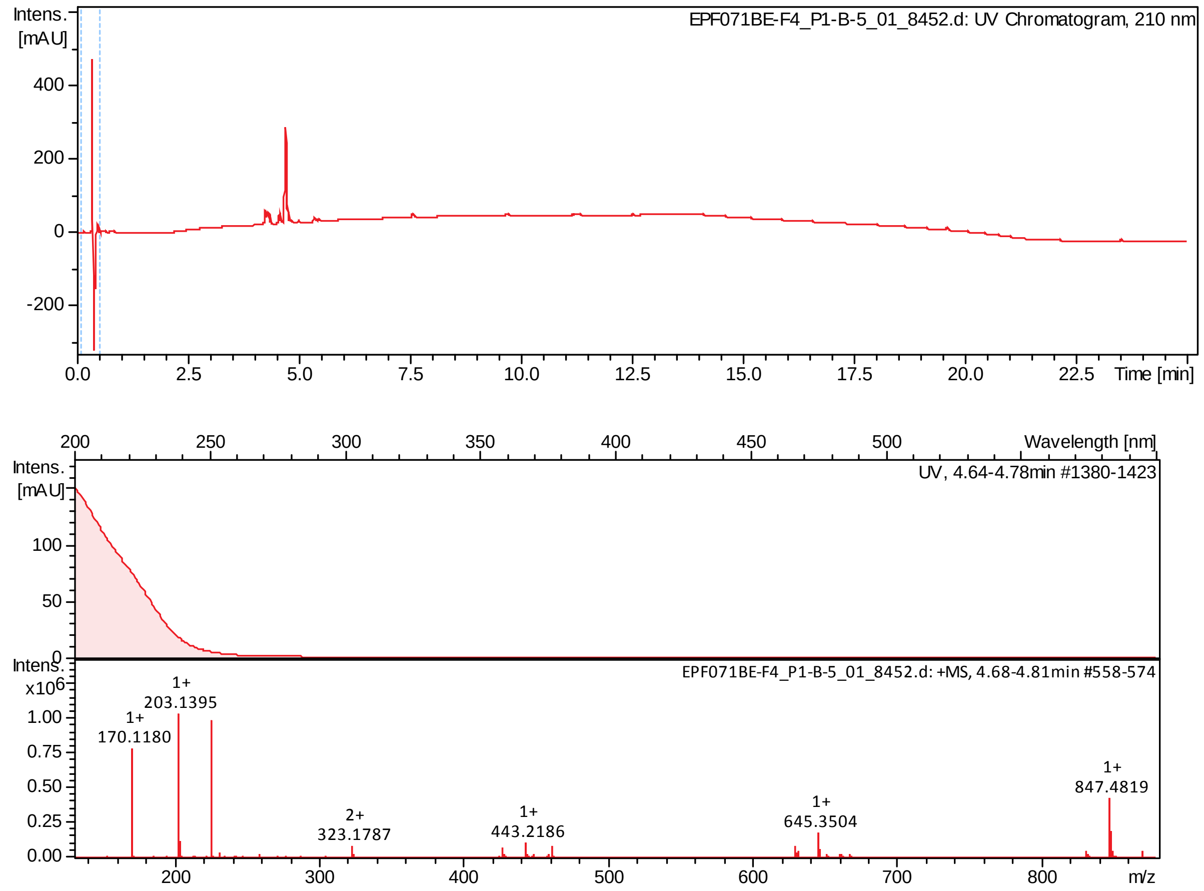

Figure S6. HR (+) ESIMS spectra of compound **5**

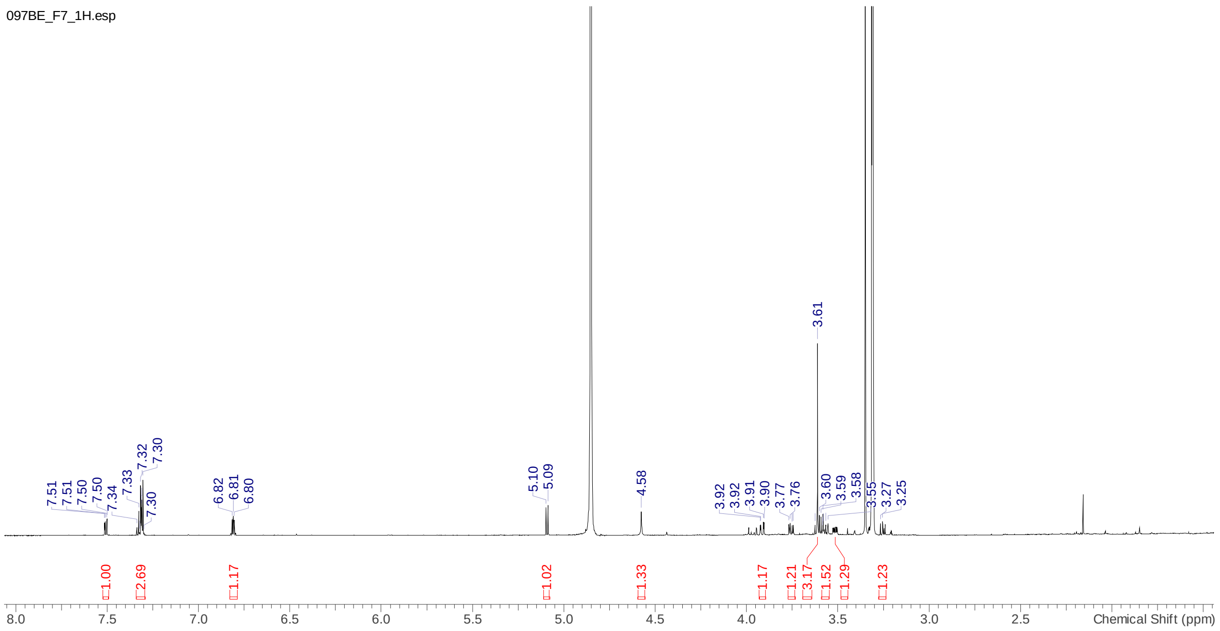

Figure S7. ^{1}H NMR spectrum for akanthol (**1**) (500 MHz, DMSO-d_6)

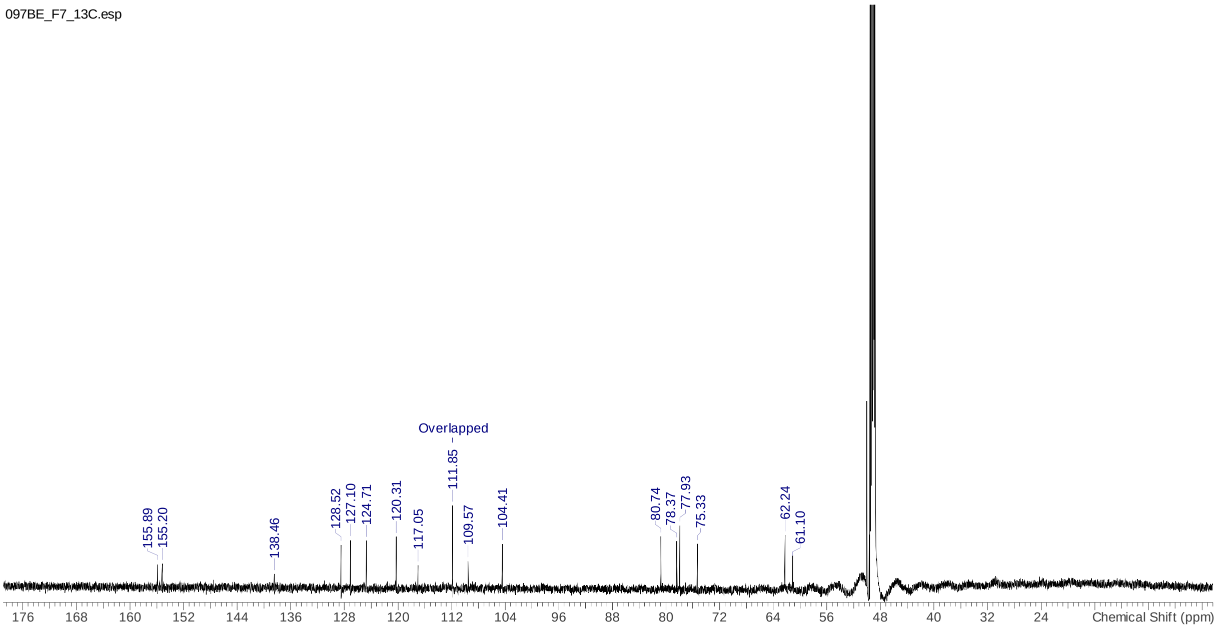

Figure S8. ^{13}C NMR spectrum for akanthol (**1**) (125 MHz, DMSO-d_6)

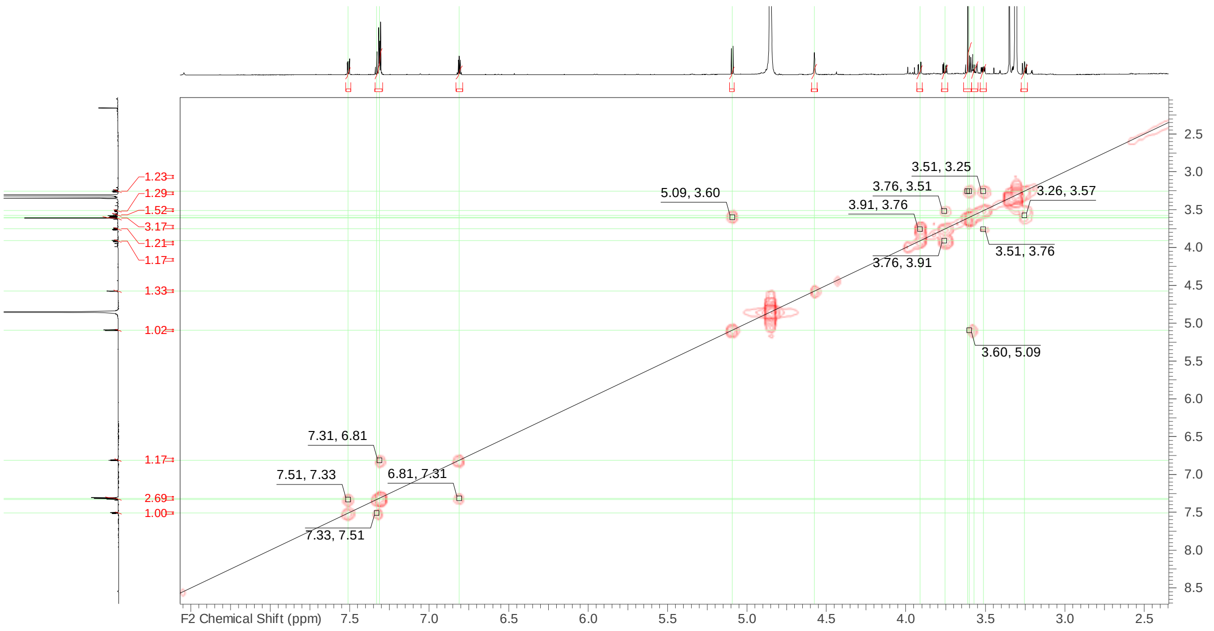

Figure S9. COSY NMR spectrum for akanthol (**1**) (500 MHz, DMSO-d_6)

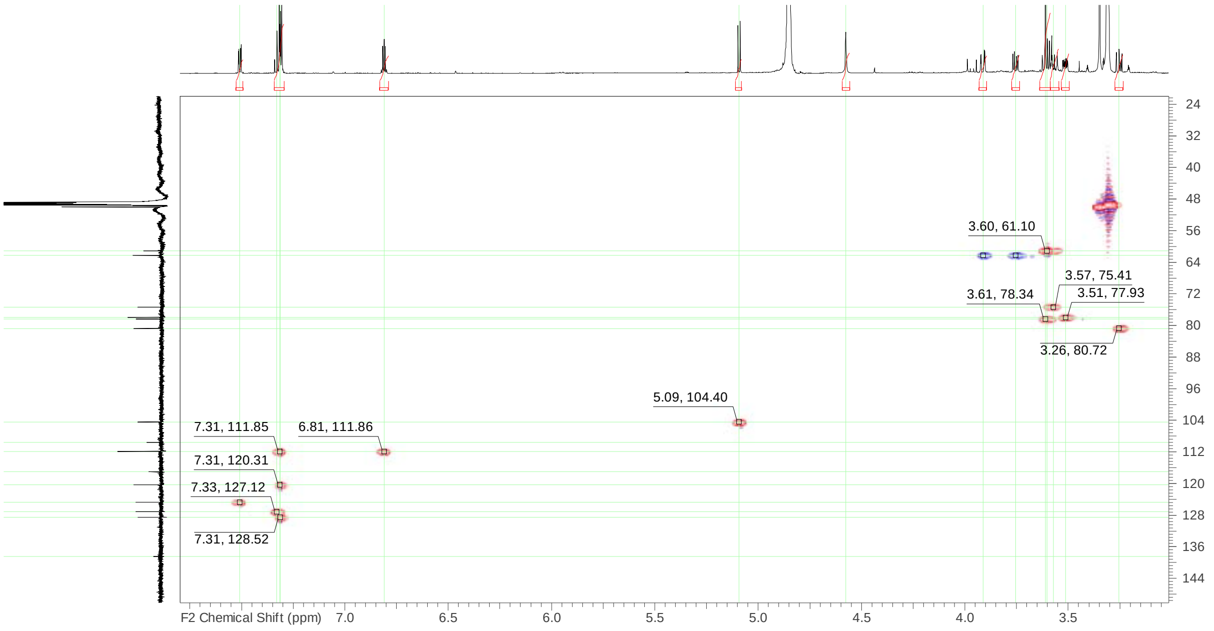

Figure S10. HSQC NMR spectrum for akanthol (**1**) (500 MHz, DMSO-d_6)

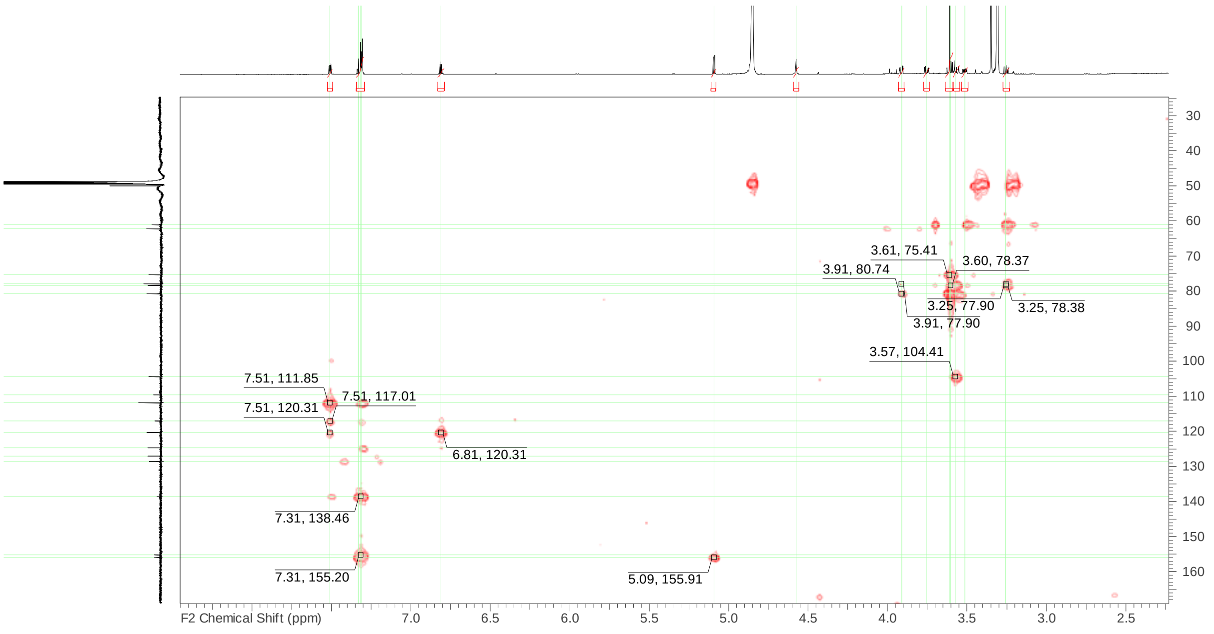

Figure S11. HMBC NMR spectrum for akanthol (**1**) (500 MHz, DMSO-d_6)

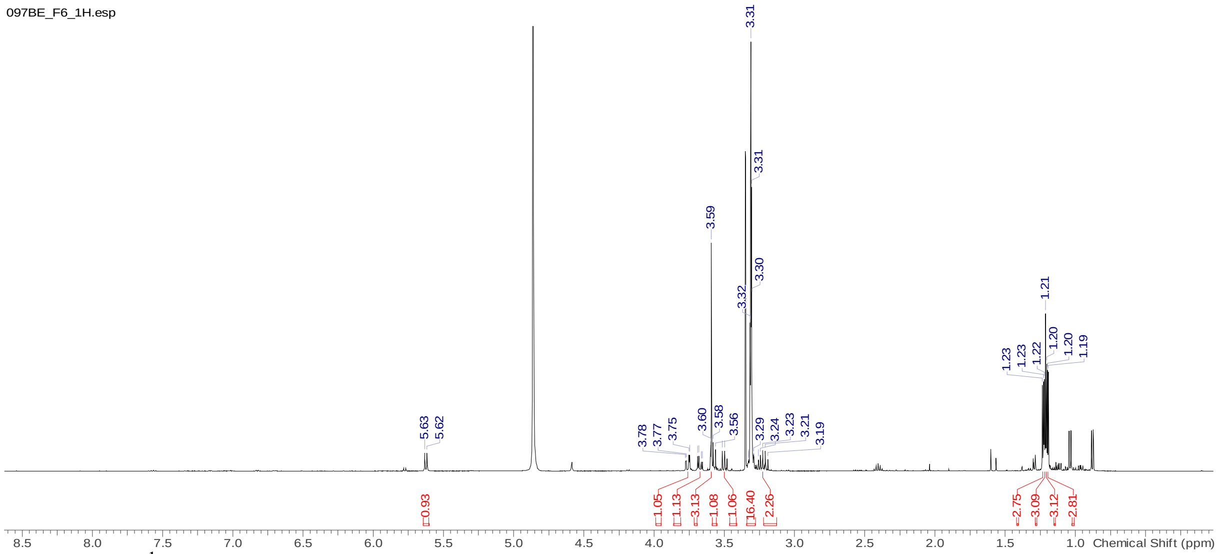

Figure S12.^{1}H NMR spectrum for akanthozine (**2**) (500 MHz, Methanol-d_4)

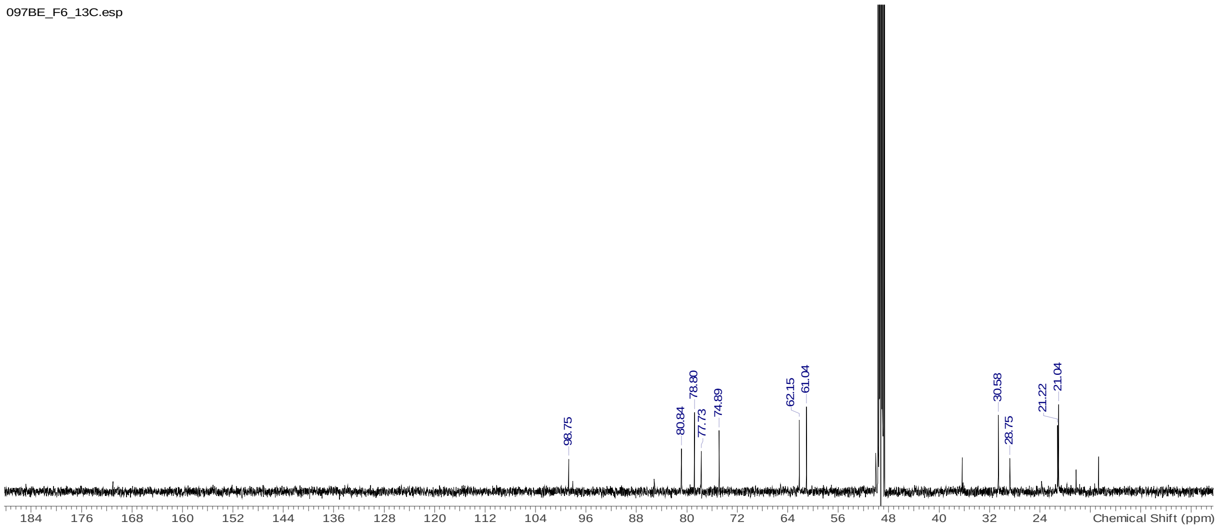

Figure S13.^{13}C NMR spectrum for akanthozine (**2**) (125 MHz, Methanol-d_4)

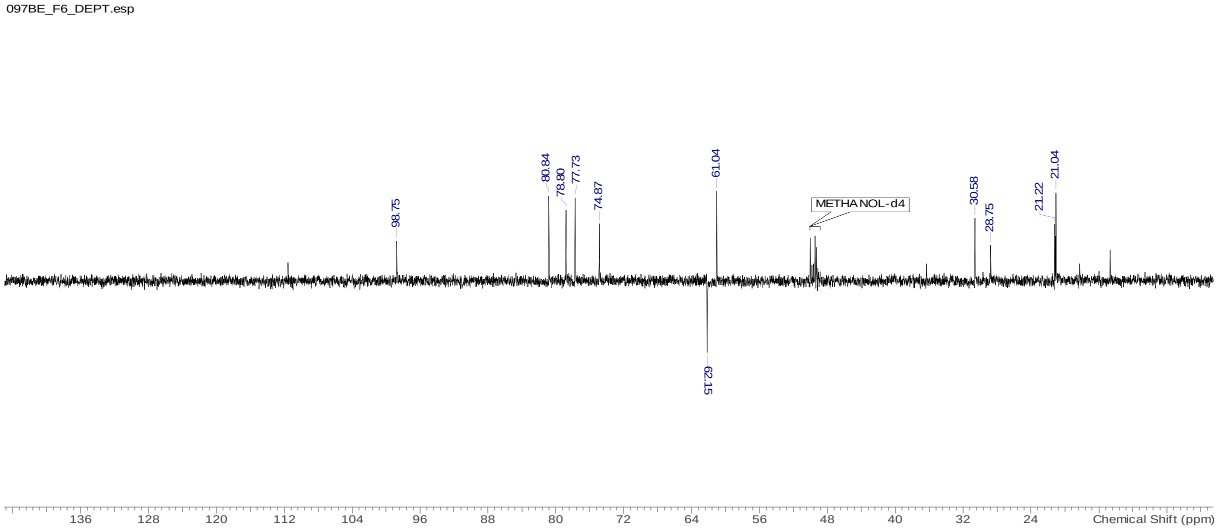

Figure S14. DEPT NMR spectrum for akanthozine (**2**) (125 MHz, Methanol-d_4)

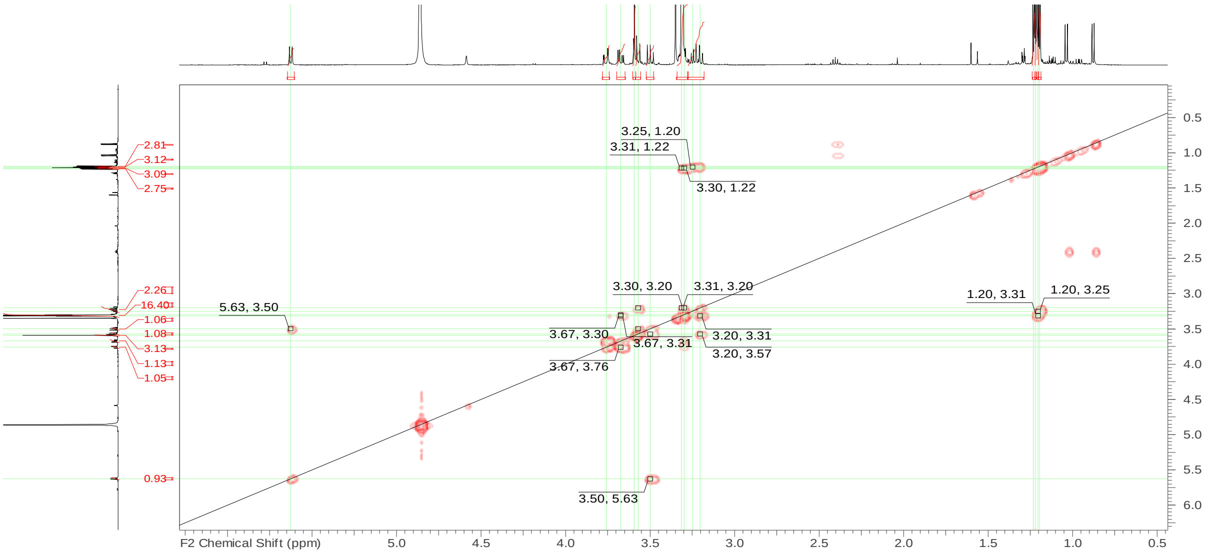

Figure S15. COSY NMR spectrum for akanthozine (**2**) (500 MHz, Methanol-d_4)

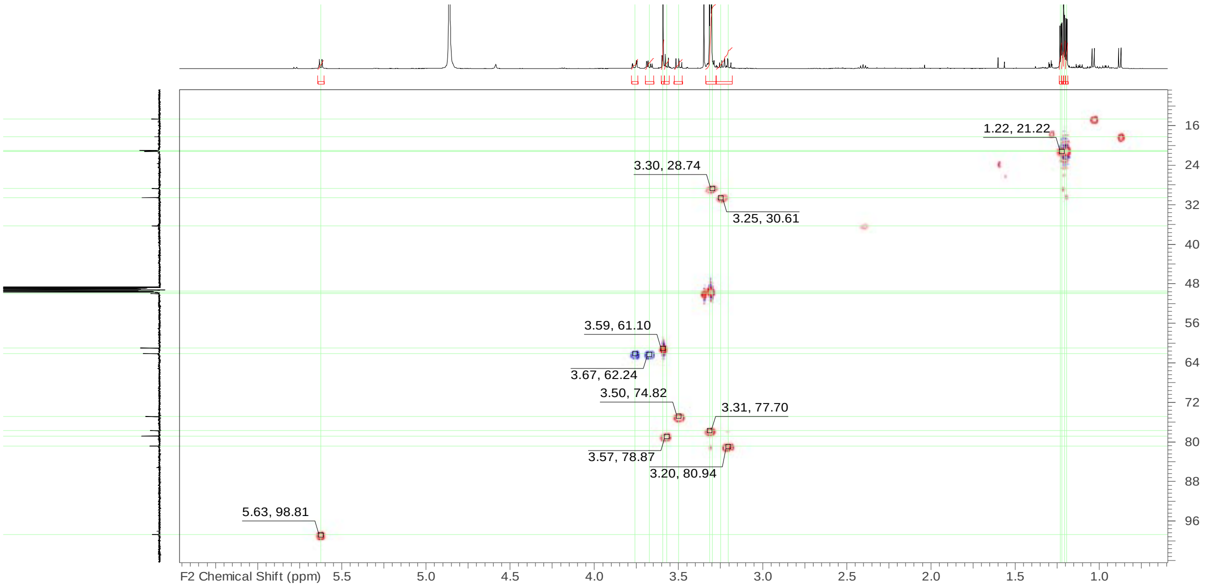

Figure S16. HSQC NMR spectrum for akanthozine (**2**) (500 MHz, Methanol-d_4)

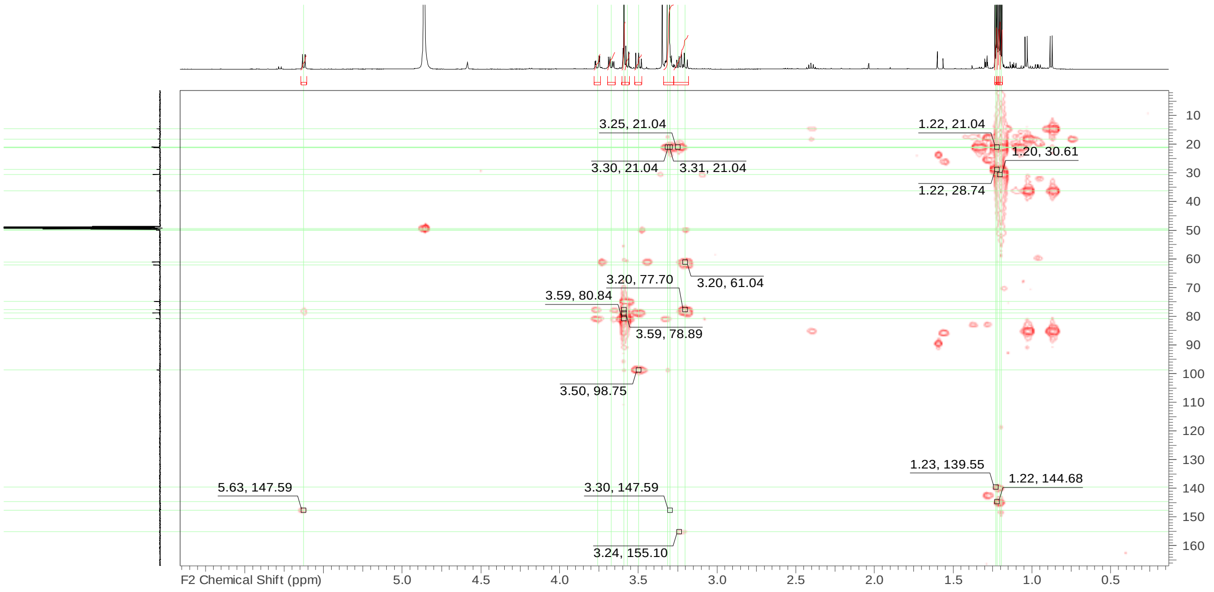

Figure S17. HMBC NMR spectrum for akanthozine (**2**) (500 MHz, Methanol-d$_4$)

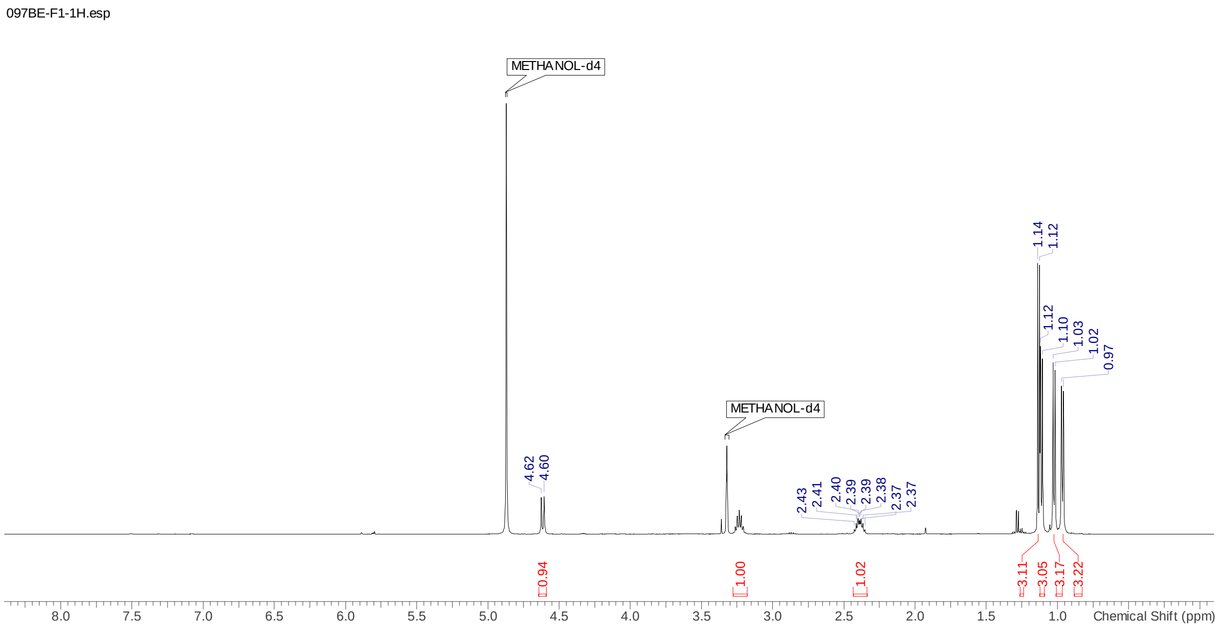

Figure S18.^{1}H NMR spectrum for compound **3** (500 MHz, Methanol-d$_4$)

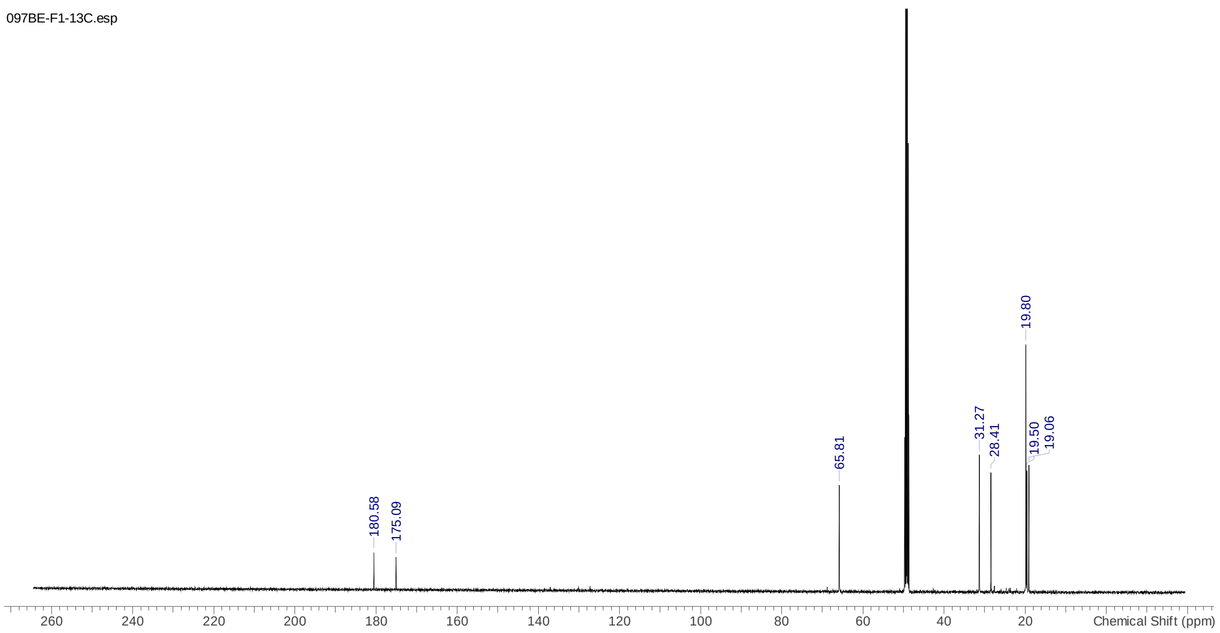

Figure S19. ^{13}C NMR spectrum for compound **3** (125 MHz, Methanol-d$_4$)

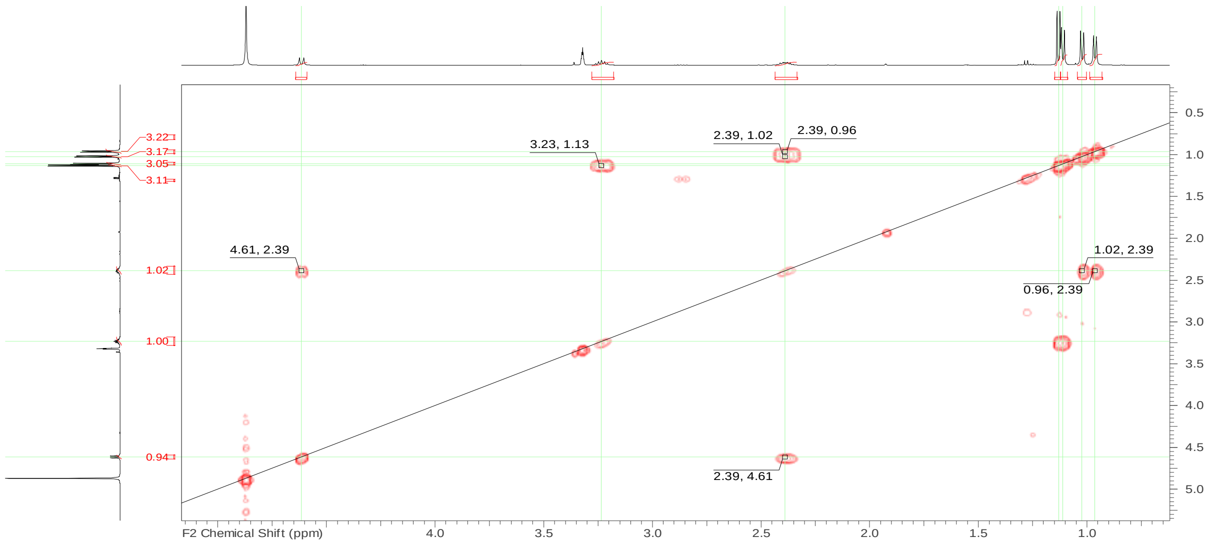

Figure S20. COSY NMR spectrum for compound **3** (500 MHz, Methanol-d$_4$)

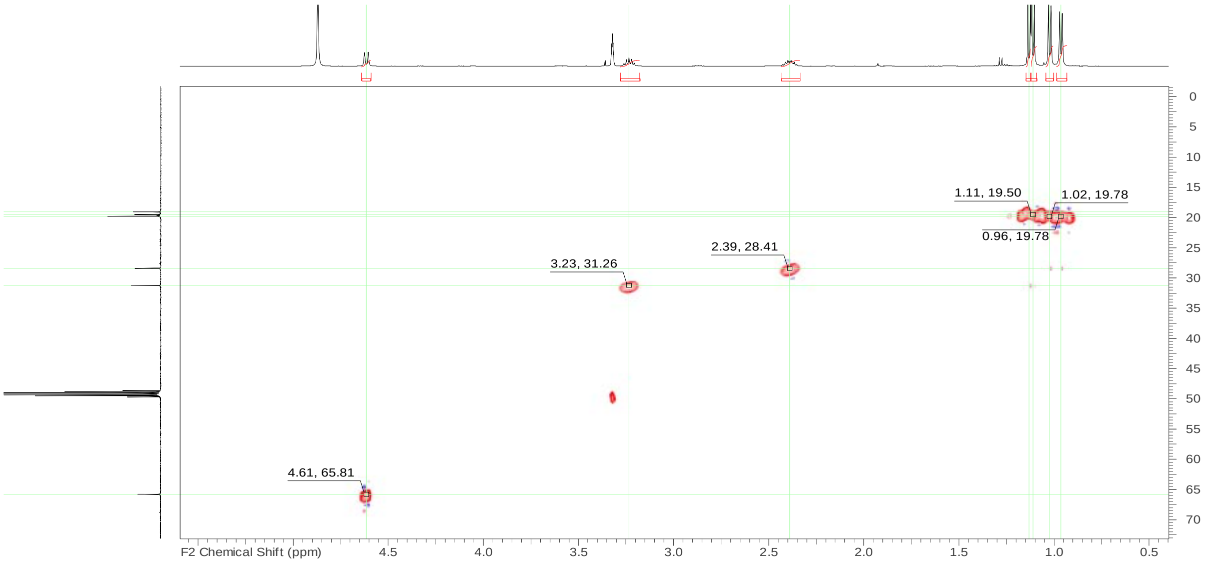

Figure S21. HSQC NMR spectrum for compound **3** (500 MHz, Methanol-d_4)

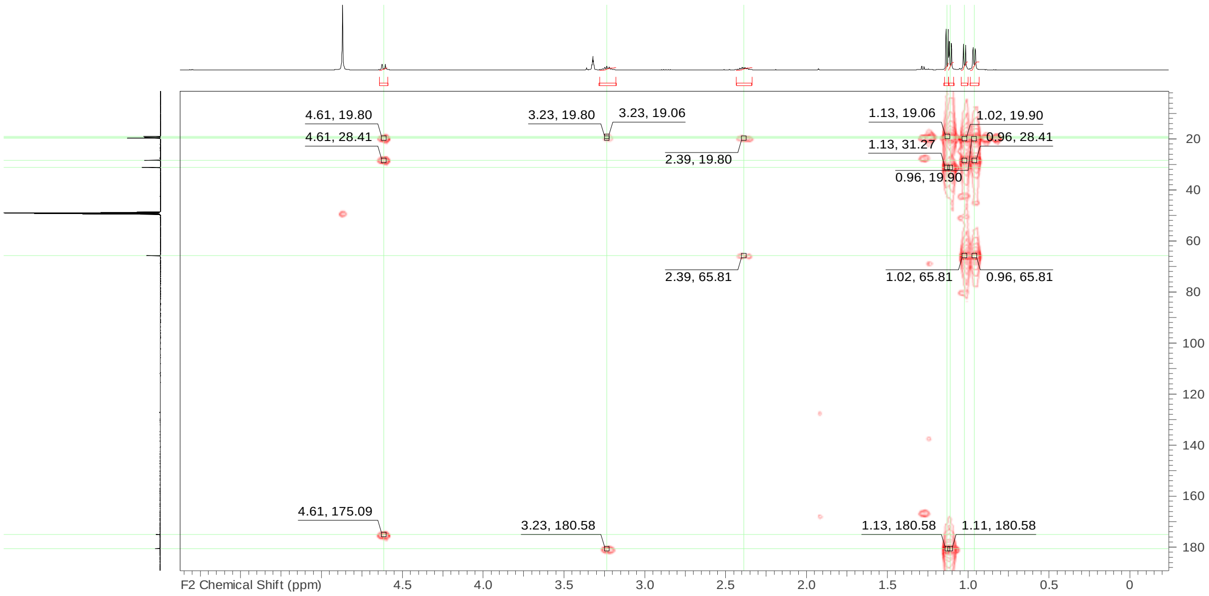

Figure S22. HMBC NMR spectrum for compound **3** (500 MHz, Methanol-d_4)

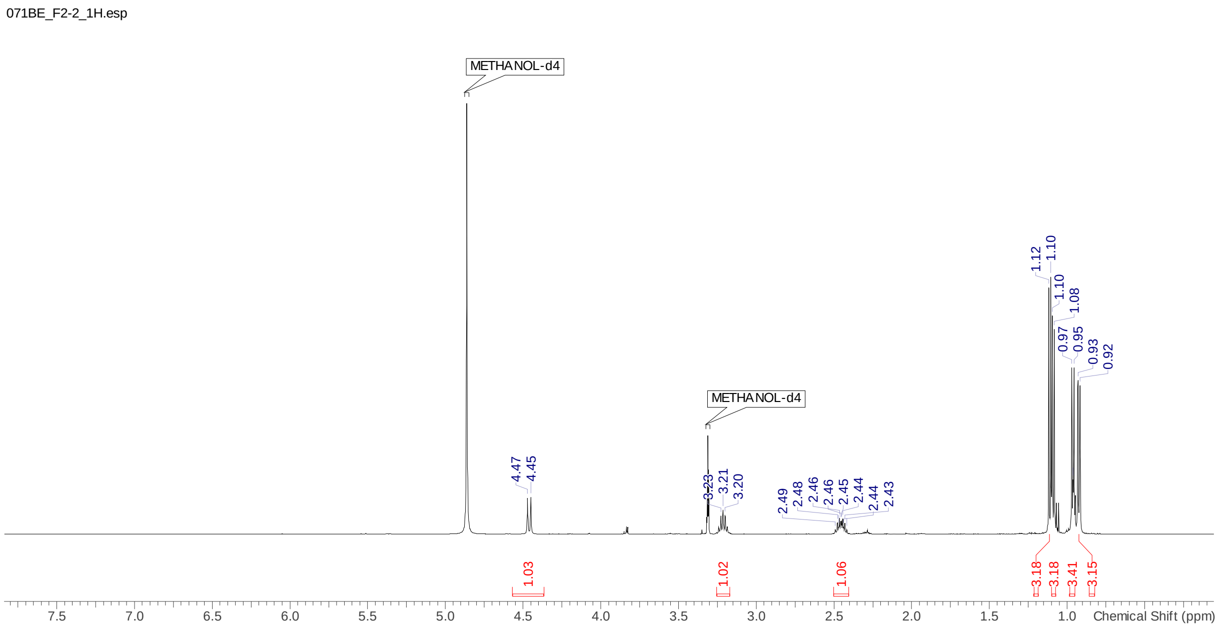

Figure S23. ^{1}H NMR spectrum for compound **4** (500 MHz, Methanol-d_4)

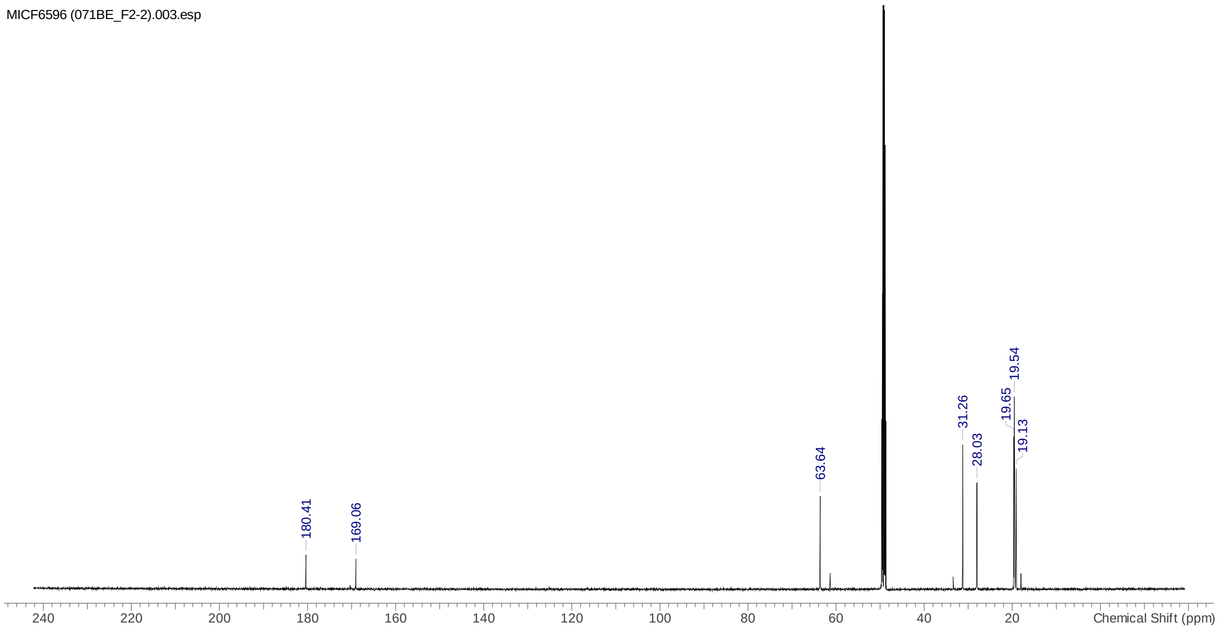

Figure S24. ^{13}C NMR spectrum for compound **4** (125 MHz, Methanol-d_4)

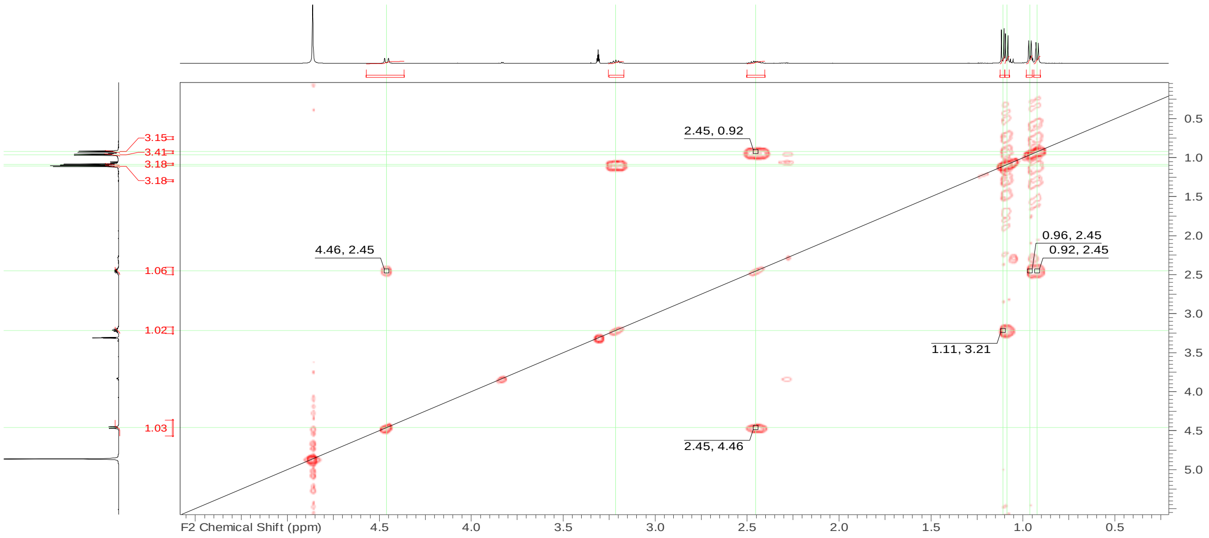

Figure S25. COSY NMR spectrum for compound **4** (500 MHz, Methanol-d_4)

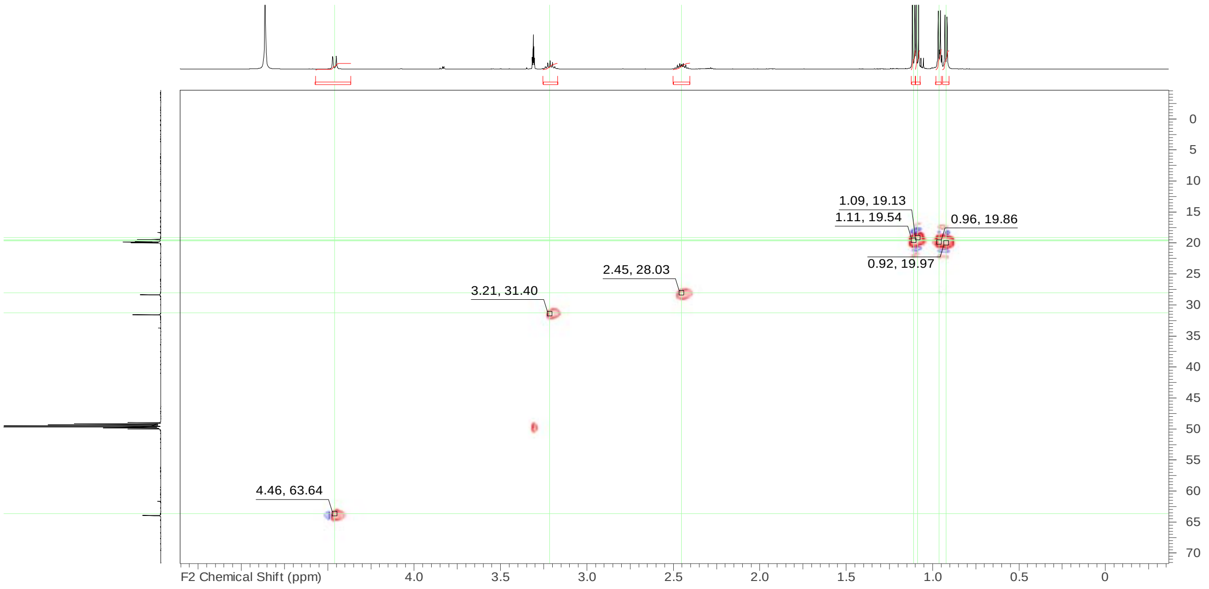

Figure S26. HSQC NMR spectrum for compound **4** (500 MHz, Methanol-d_4)

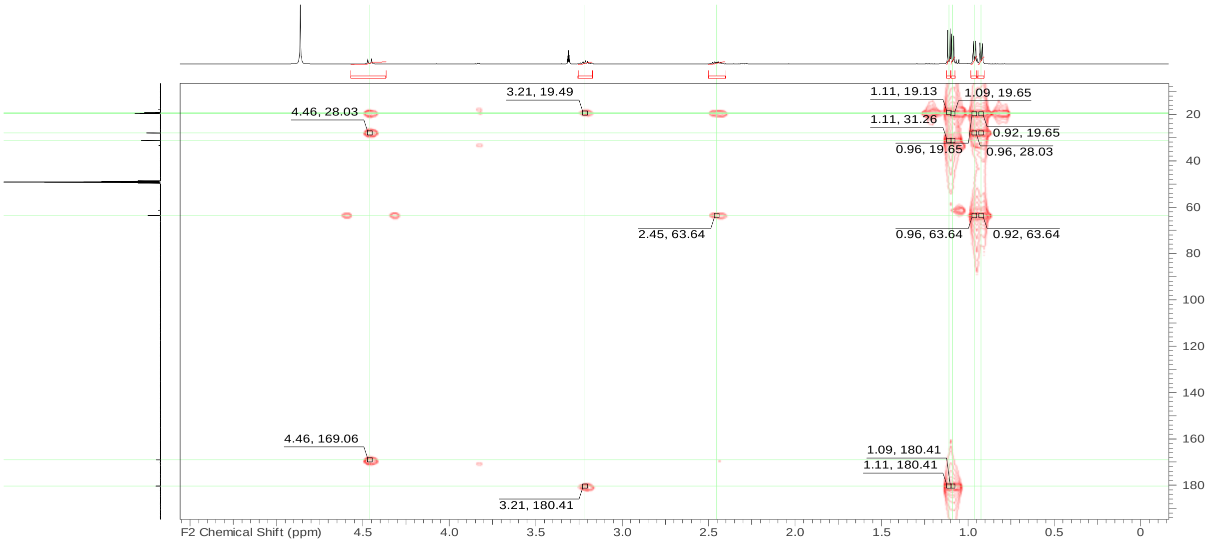

Figure S27. HMBC NMR spectrum for compound **4** (500 MHz, Methanol-d$_4$)

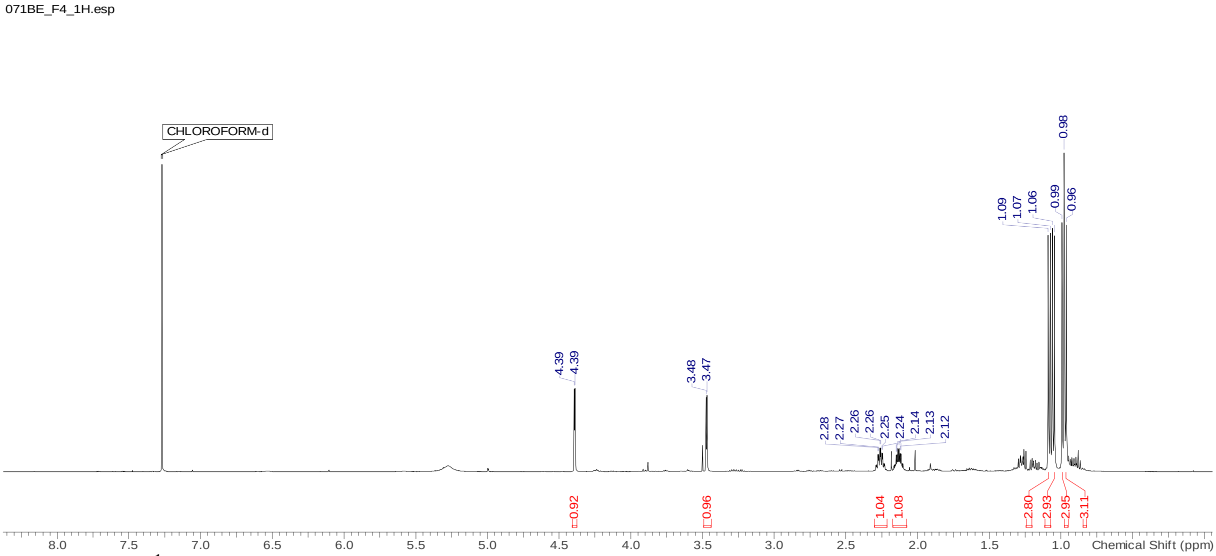

Figure S28. ^{1}H NMR spectrum for compound **5** (500 MHz, $CDCl_3$)

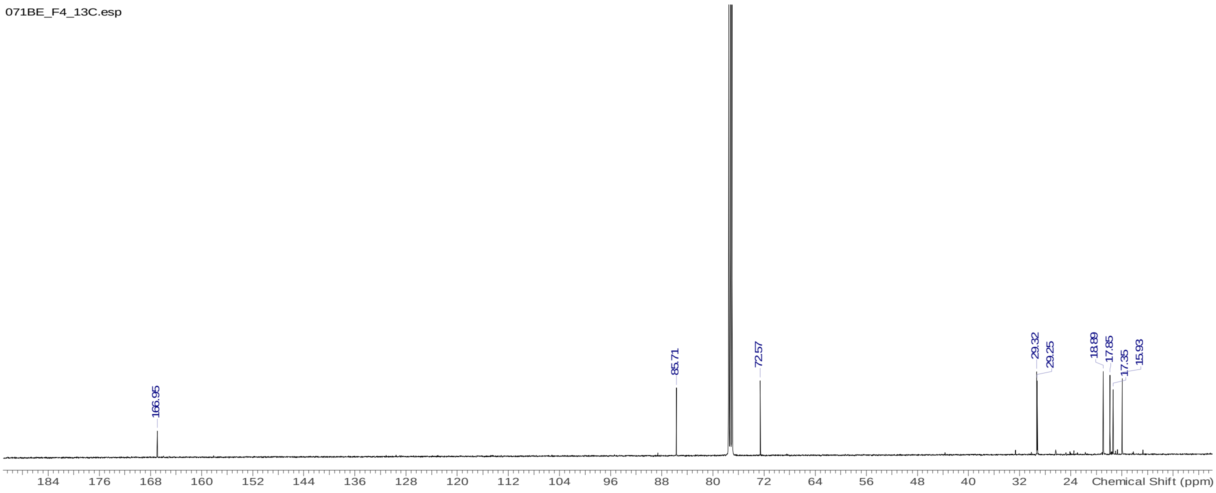

Figure S29. ^{13}C NMR spectrum for compound **5** (125 MHz, $CDCl_3$)

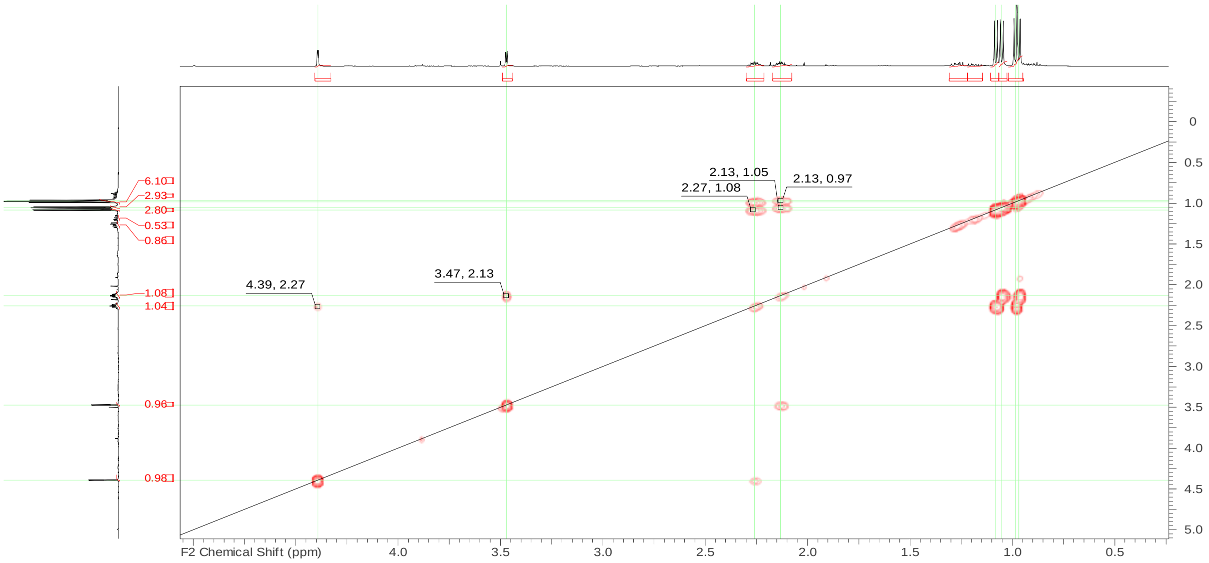

Figure S30. COSY NMR spectrum for compound **5** (500 MHz, $CDCl_3$)

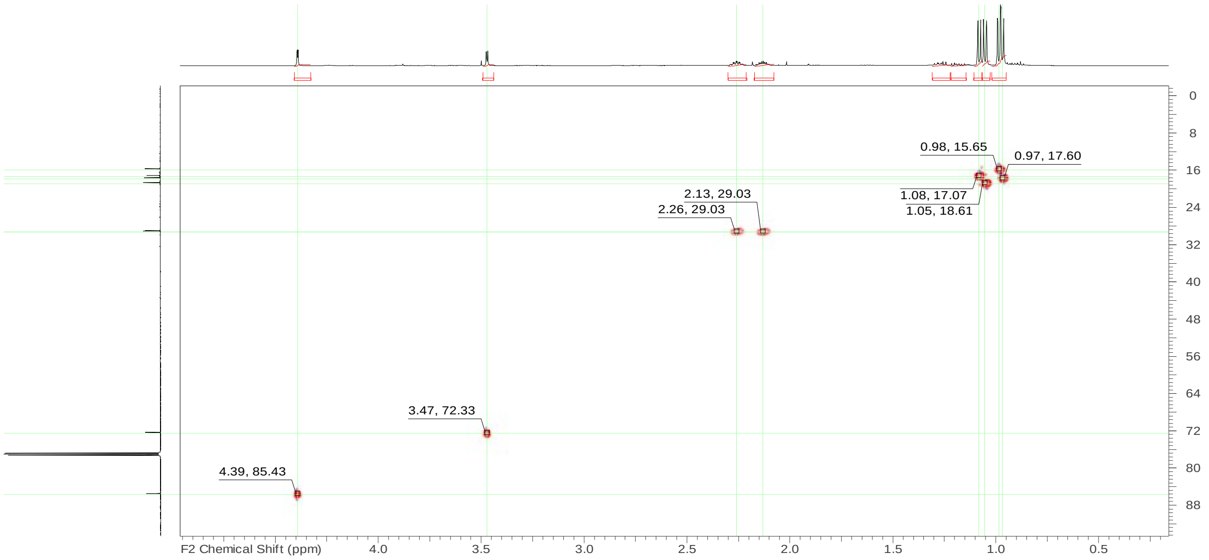

Figure S31. HSQC NMR spectrum for compound **5** (500 MHz, $CDCl_3$)

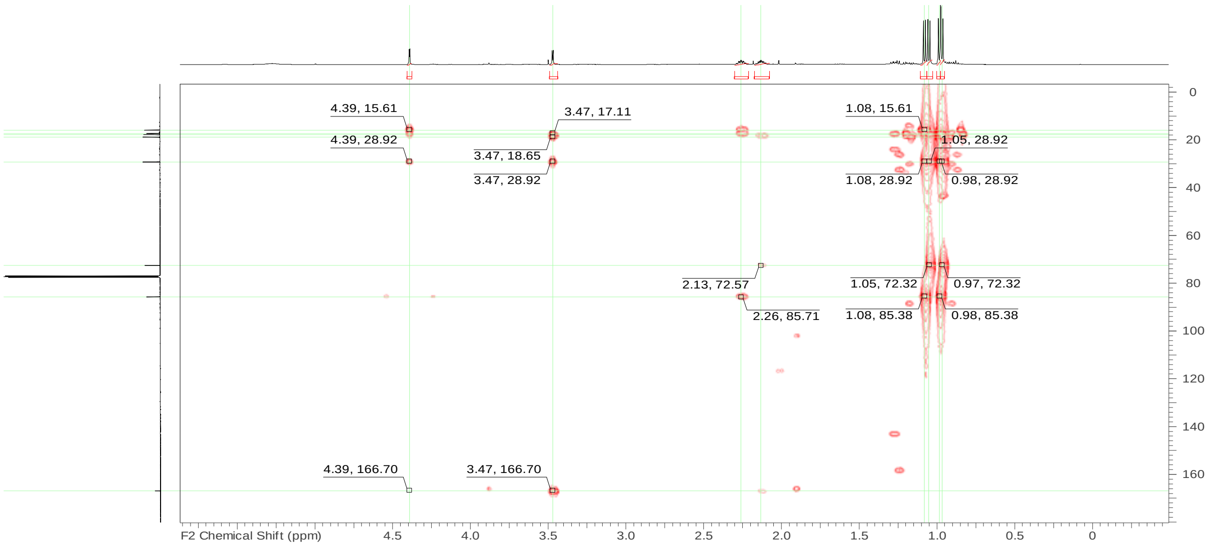

Figure S32. HMBC NMR spectrum for compound **5** (500 MHz, $CDCl_3$)

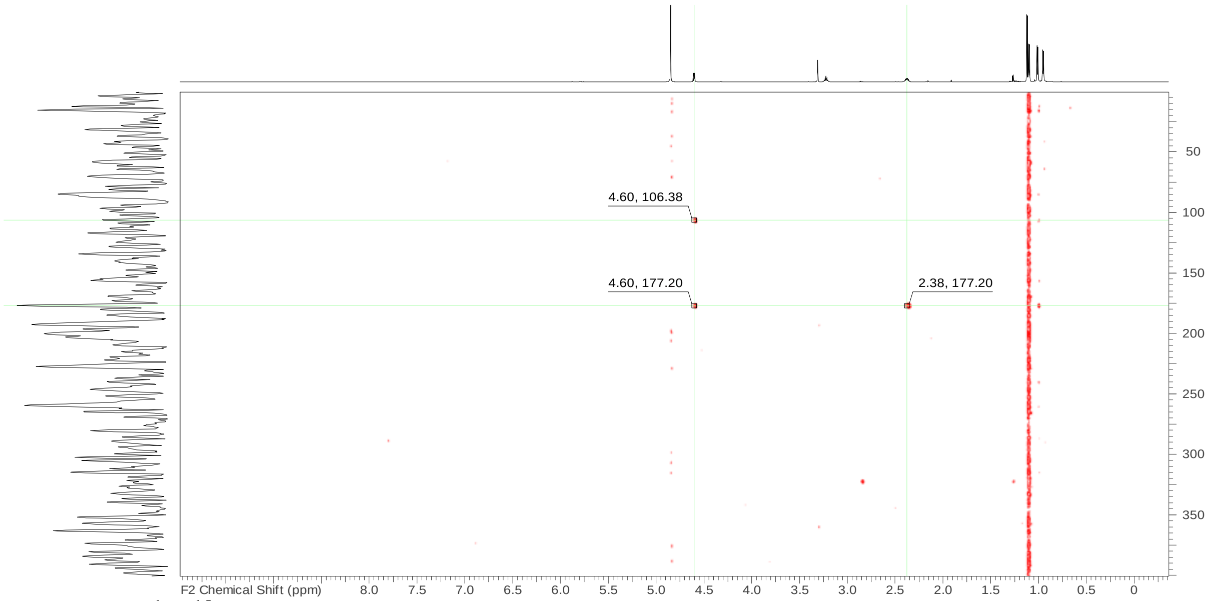

Figure S33. ^{1}H-^{15}N-HMBC NMR spectrum for compound **5** (700 MHz, $CDCl_3$)
